# COVID-19 Vaccination and Cardiopulmonary Events After Acute Coronary Syndromes: A Secondary Analysis of a Randomized Clinical Trial

**DOI:** 10.1001/jamanetworkopen.2024.13946

**Published:** 2024-05-30

**Authors:** Henrique Andrade R. Fonseca, Lucas Petri Damiani, Frederico Monfardini, André Zimerman, Luiz Vicente Rizzo, Otávio Berwanger

**Affiliations:** 1 Instituto Israelita de Ensino e Pesquisa, Hospital Israelita Albert Einstein, São Paulo, Brazil; 2 Thrombolysis in Myocardial Infarction Study Group, Brigham and Women’s Hospital, Harvard Medical School, Boston, Massachusetts; 3 George Institute for Global Health UK at Imperial College, London, United Kingdom

## Abstract

This secondary analysis of a randomized clinical trial investigates the association of COVID-19 vaccination with incidence of cardiopulmonary events among patients who had experienced acute coronary syndromes.

## Introduction

COVID-19 vaccines effectively reduce the risk of hospitalizations and death.^1,2^ However, the efficacy of COVID-19 vaccines in reducing the incidence of cardiopulmonary events in a secondary prevention population at high risk of cardiovascular events remain unstudied, to our knowledge.

## Methods

This secondary analysis of a randomized clinical trial was approved by the Comitê de Ética em Pesquisa-Hospital Israelita Albert Einstein, and all participants provided informed written consent before participation. The study followed the CONSORT reporting guideline.

We conducted a prespecified analysis of the Vaccination Against Influenza to Prevent Cardiovascular Events After Acute Coronary Syndromes (VIP-ACS) trial.^3^ This randomized, multicenter trial assessed the efficacy of the influenza vaccination strategy after an ACS between July 19, 2019, and November 30, 2020. In this prespecified analysis, we compared the incidence of cardiopulmonary events in patients who received vs did not receive COVID-19 vaccination in Brazil. Patients were not randomized to COVID-19 vaccines.

The primary end point consisted of all-cause death, myocardial infarction, stroke, hospitalization for unstable angina, hospitalization for heart failure, urgent coronary revascularization, or hospitalization for respiratory infections. The secondary end point consisted of major adverse cardiovascular events (MACE). Trial design details can be found in the trial protocol in Supplement 1 and eMethods in Supplement 2.

Patients who received at least 1 dose of a COVID-19 vaccine during follow-up were classified as COVID-19 vaccinated at the time of the vaccine and subsequently censored from the unvaccinated group.^3^ To minimize ascertainment immortal bias, we applied a landmark analysis excluding patients who had any end points within 90 days of randomization, as suggested by Fu et al.^4^

End points were analyzed using adjusted Cox proportional hazard models. A 2-tailed *P* < .05 was considered significant. Analyses were performed using R statistical software version 4.2.0 (R Project for Statistical Computing).

## Results

Among 1801 participants (median [IQR] age, 56.7 [50.1-64.5] years; 541 females [30.3%]), 292 individuals (16.2%) had a prior myocardial infarction and 35.7% were active smokers. A total of 1665 individuals did not have cardiopulmonary events during the first 90 days and were included in the primary analysis, among whom 835 individuals (50.2%) had taken at least 1 COVID-19 vaccine dose. Most individuals (63.9%) received at least 1 viral vector vaccine (Oxford/AstraZeneca [ChAdOx1]) dose during follow-up.

In the 90-day event-free follow-up analysis of unvaccinated individuals, the incidence of the primary end point per 100 patient-years was 9.37 vs 4.81 events for those who had received at least 1 vaccine dose (adjusted hazard ratio [aHR], 0.41; 95% CI, 0.18-0.94; *P* = .12) (Figure, A). Vaccination did not significantly reduce incidence of MACE (aHR, 0.32; 95% CI, 0.07-1.53; *P* = .60) (Figure, B), all-cause death (aHR, 0.29; 95% CI, 0.09-0.91; *P* = .12) (Figure, C), or cardiovascular death (aHR, 0.42; 95% CI, 0.04-4.02; *P* > .99) (Figure, D). A sensitivity analysis of all participants revealed similar findings for the adjusted incidence of the primary composite end point (aHR, 0.43; 95% CI, 0.02-0.94; *P* = .12) and all-cause death (aHR, 0.30; 95% CI, 0.10-0.89; *P* = .12) (Table).

**Figure.**
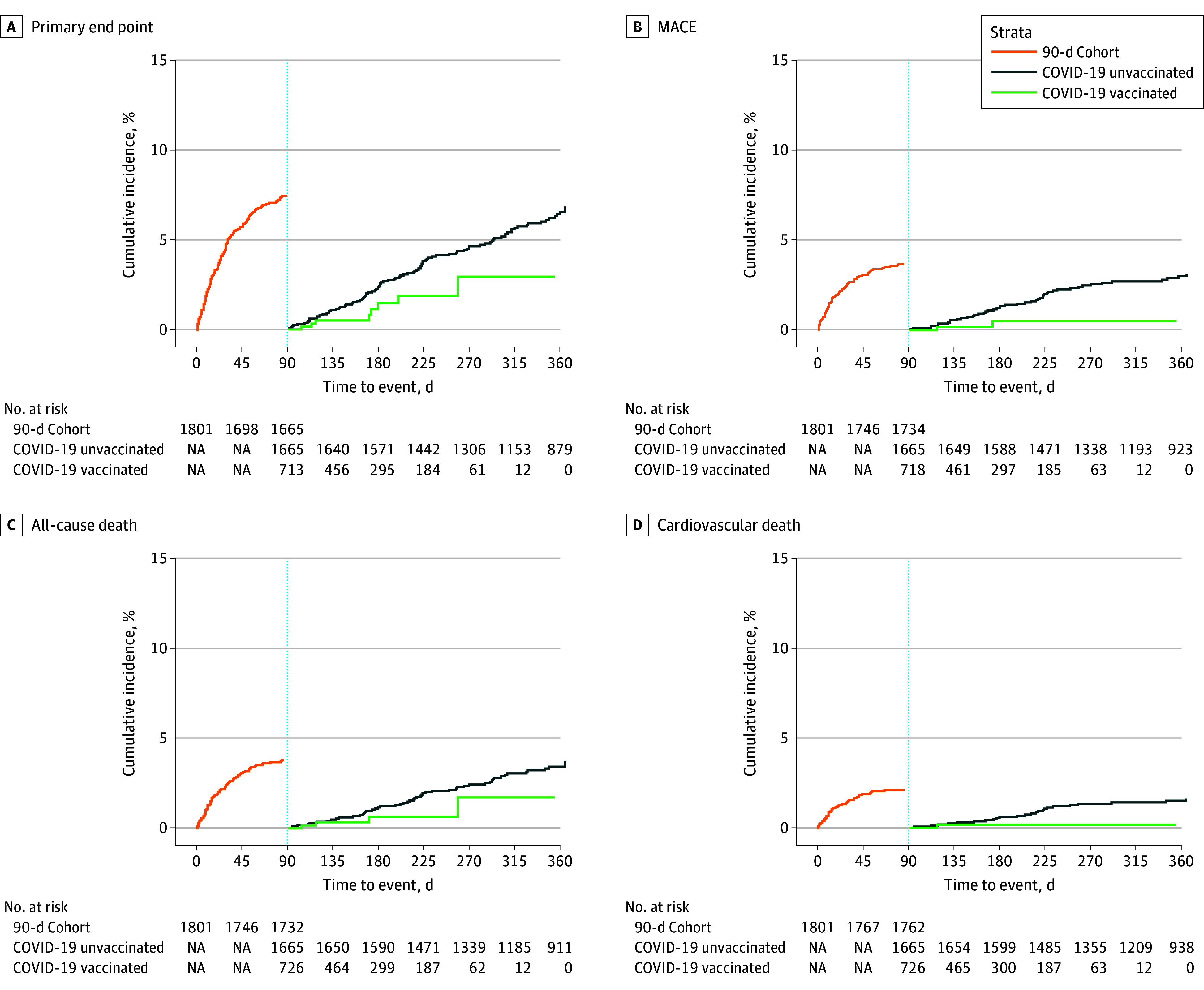
Kaplan-Meier Estimates for Primary and Secondary End Points The COVID-19 vaccine date was modeled as a time-dependent covariable in a Cox model adjusted for age, allocation group, and natural splines for randomization date to account for seasonal peaks due to the pandemic. Kaplan-Meir event curves include all randomized patients in the Vaccination Against Influenza to Prevent Cardiovascular Events After Acute Coronary Syndromes trial. A, Event curves for the primary end point (a composite of all-cause death, myocardial infarction, stroke, hospitalization for unstable angina, hospitalization for heart failure, urgent coronary revascularization, or hospitalizations for respiratory infection) are shown. B, Event curves for major adverse cardiovascular events (MACE; cardiovascular death, myocardial infarction, and stroke) are shown. C, Event curves for all-cause death are shown. D, Event curves for cardiovascular death are shown.

**Table.** Event Rates and Estimated HRs After First COVID-19 Vaccine Dose

End point	Events, No./100 patient-y			HR (95% CI)			
	Patients with 90-d follow-up^a^	90-d Event-free population					
		Unvaccinated	Vaccinated	90-d Event-free population^b^	*P* value^c^	Full population^d^	*P* value
Primary end point	32.30	9.37	4.81	0.41 (0.18-0.94)	.12	0.43 (0.20-0.94)	.12
MACE	15.74	4.32	1.19	0.32 (0.07-1.53)	.60	0.27 (0.06-1.20)	.32
CV death	8.93	2.23	0.59	0.42 (0.04-4.02)	>.99	0.27 (0.03-2.16)	.84
All-cause death	15.97	4.98	2.37	0.29 (0.09-0.91)	.12	0.30 (0.10-0.89)	.12
Follow up, mean (SD), d	335.6 (56.9)	349.5 (52.0)	321.8 (61.8)	NA	NA	NA	NA

Abbreviations: CV, cardiovascular; HR, hazard ratio; MACE, major adverse cardiovascular events; NA, not applicable.

^a^ Follow-up within 90 days.

^b^ This model applied a Cox proportional hazard regression including 1665 patients from the 90-day event-free population using COVID-19 vaccination as a time-dependent covariable adjusted for age, allocation group (standard-dose and double-dose influenza vaccine), and natural splines for randomization date to account for seasonal peaks due to the COVID-19 pandemic.

^c^ *P* value adjusted for multiple comparison (*P* < .01).

^d^ This model applied a Cox proportional hazard regression including 1801 patients (full population) using COVID-vaccines as a time-dependent covariable adjusted for age, allocation group (standard-dose and double-dose influenza vaccine), and natural splines for randomization date to account for seasonal peaks due to the COVID-19 pandemic.

## Discussion

In this secondary analysis of a randomized clinical trial, patients who received at least 1 COVID-19 vaccine dose after ACS had similar rates of the primary composite end point and MACE compared with unvaccinated patients. However, retrospective studies^5,6^ have demonstrated a short-term reduction in MACE risk after COVID-19 vaccination.

Major strengths of this study were findings derived from multicenter, blinded adjudication of an end points trial. Limitations include the prespecified exploratory analysis design, insufficient statistical power, and lack of randomization for COVID-19 vaccines. Therefore, residual confounding or unmeasured variables could explain the findings. Our results should not be generalized to other COVID-19 vaccines.

## Notes

Supplement 1.
eMethods.

eReferences.

Supplement 2.
Trial Protocol and Statistical Analysis Plan

Supplement 3.
Data Sharing Statement
