# Supplementary material for: COVID-19 Vaccination and Cardiopulmonary Events After Acute Coronary Syndromes: A Secondary Analysis of a Randomized Clinical Trial
Source: JAMA Netw Open. 2024 May 30;7(5):e2413946. doi: 10.1001/jamanetworkopen.2024.13946 (PMC11140521; doi:10.1001/jamanetworkopen.2024.13946)
Supplement: Supplement 1. — eMethods. eReferences. [file jamanetwopen-e2413946-s001.pdf]

Supplemental Online Content

Fonseca HAR, Damiani LP, Monfardini F, Zimerman A, Rizzo LV, Berwanger O. COVID-19 vaccination and cardiopulmonary events after acute coronary syndromes: a secondary analysis of a randomized clinical trial. *JAMA Netw Open*. 2024;7(5):e2413946. doi:10.1001/jamanetworkopen.2024.13946

eMethods.

eReferences.

This supplemental material has been provided by the authors to give readers additional information about their work.

© 2024 Fonseca HAR et al. *JAMA Network Open.*

eMethods

COVID-19 vaccines delivery in Brazil:

Oxford-Astrazeneca (ChAdOx1), COVID-19 replication-deficient adenoviral vaccine.

CoronaVac-Sinovac/Butantã, inactivated whole-virion COVID-19 vaccine

Pfizer-BioNTech (BNT162b2), COVID-19mRNA vaccine.

Janssen (Ad26.COV2.S), COVID-19 replication-deficient adenoviral vaccine.

VIP-ACS trial

The VIP-ACS was a pragmatic, randomized, multicenter, active-comparator, open-label trial with blinded outcome adjudication comparing two strategies of influenza vaccination following an acute coronary syndrome (ACS). The COVID-19 cases, and vaccines reporting was mandatory during follow-up. Adults aged 18 years or older after an ACS were included between July 2019 and November 2020, detailed were previously published [1].

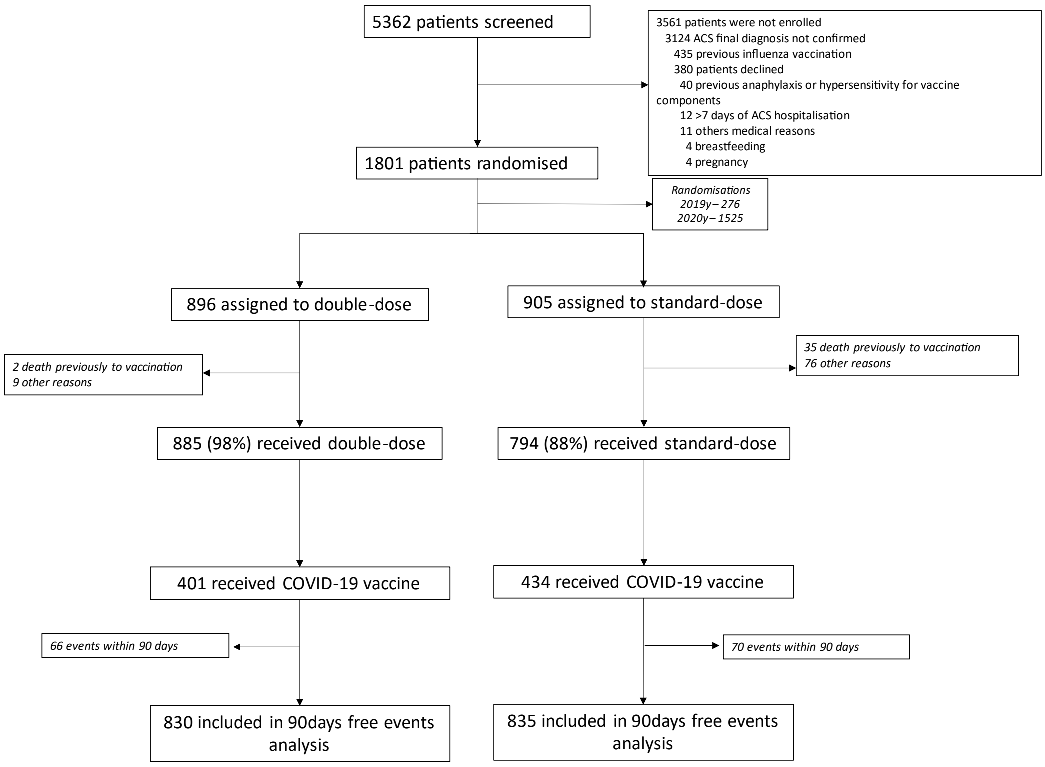

CONSORT - VIP-ACS Trial

Trial End points

All study end points and causes of death were adjudicated using standardized definitions by an independent clinical events committee (CEC), whose members were unaware of randomize

© 2024 Fonseca HAR et al. *JAMA Network Open.*

treatment assignments. Detailed end point adjudication is provided in previously published VIP-ACS study [1].

Statistical analysis

The initial 90 days post-ACS are critical, with a recurrence of events being particularly pronounced; 28% of patients face readmission within this period, many of which occur between 30 and 90 days [2]. To minimize ascertainment bias, the analyses excluded patients who had presented with the study end points or had died within 90 days after randomization. We performed a landmark analysis for a 90-day event-free period, comparing vaccinated to unvaccinated participants. The event date was input as time-varying covariables in the Cox proportional hazard model [3].

Primary and secondary end points were analyzed using COVID vaccine as a time dependent covariable, adjusted for age, allocation group (standard and doble-dose influenza vaccine), and natural splines for randomization date to account for seasonal peaks due to pandemic. The data are presented as point estimates and 95% confidence interval (CI) with two-sided P-value. The p value was adjusted for multiple comparations (<0.01).

© 2024 Fonseca HAR et al. *JAMA Network Open.*

eReferences

1. Fonseca HA, Furtado RH, Zimerman A, Lemos PA, Franken M, Monfardini F, et al., Influenza vaccination strategy in acute coronary syndromes: the VIP-ACS trial. Eur Heart J. 2022; 43(41):4378-4388. doi: 10.1093/eurheartj/ehac472.
2. Kini V, Peterson PN, Spertus JA, Kennedy KF, Arnold SV, Wasfy JH, Curtis JP, Bradley SM, Amin AP, Ho PM, Masoudi FA. Clinical Model to Predict 90-Day Risk of Readmission After Acute Myocardial Infarction. Circ Cardiovasc Qual Outcomes. 2018;11(10):e004788. doi: 10.1161/CIRCOUTCOMES.118.004788.
3. Fu EL, van Diepen M, Xu Y, Trevisan M, Dekker FW, Zoccali C, Jager K, Carrero JJ. Pharmacoepidemiology for nephrologists (part 2): potential biases and how to overcome them. Clin Kidney J. 2020;14(5):1317-1326. doi: 10.1093/ckj/sfaa242.

© 2024 Fonseca HAR et al. *JAMA Network Open.*
