# Supplementary material for: COVID-19 Vaccination and Cardiopulmonary Events After Acute Coronary Syndromes: A Secondary Analysis of a Randomized Clinical Trial
Source: JAMA Netw Open. 2024 May 30;7(5):e2413946. doi: 10.1001/jamanetworkopen.2024.13946 (PMC11140521; doi:10.1001/jamanetworkopen.2024.13946)
Supplement: Supplement 2. — Trial Protocol and Statistical Analysis Plan [file jamanetwopen-e2413946-s002.pdf]

ALBERT EINSTEIN
SOCIEDADE BENEFICENTE ISRAELITA BRASILEIRA

Evaluation of the Effectiveness of Double Dose Influenza Vaccination to Reduce Major Cardiovascular Events After an Acute Coronary Syndrome

***V**accination against **I**nfluenza to **P**revent cardiovascular events after **A**cute **C**oronary **S**yndrome*

VIP-ACS

Steering Committee: Otávio Berwanger (Chair, Principal Investigator)
Henrique Andrade R. Fonseca (Principal Investigator)
Remo Holanda M. Furtado
Hélio Penna Guimarães
Adriano Mendes Caixeta
Pedro Lemos
Marcelo Franken
José Carlos Nicolau

Coordinating Site: Academic Research Organization (ARO) - Hospital Israelita Albert Einstein (HIAE)
Sociedade Beneficente Israelita Brasileira Hospital Albert Einstein
Avenida Albert Einstein, 627, Bloco A, 2º subsolo, Jardim Leonor, São Paulo – SP - Brasil

Version: 6.0 – October/2021

Description of protocol versions is found on Appendix A.

This document is confidential, and its use, reproduction, disclosure and publication are restricted to the principal investigators and the sponsor.

Sponsor: PROADI-SUS – NUP:25000.030761/2018-54
Protocol: Evaluation of the Effectiveness of the Vaccination Against Influenza to Reduce Mortality and Burden in Chronic Diseases (VIP Study)

ALBERT EINSTEIN
SOCIEDADE BENEFICENTE ISRAELITA BRASILEIRA

**Title**	***Evaluation of the Effectiveness of Double Dose Influenza Vaccination to Reduce Major Cardiovascular Events After an Acute Coronary Syndrome - VIP-ACS***<br>***"Vaccination against Influenza to Prevent cardiovascular events after Acute Coronary Syndrome - VIP-ACS"***
**Project Main Office**	Academic Research Organization-<br>Hospital Israelita Albert Einstein<br>Avenida Albert Einstein, 627, Bloco A, 2º subsolo, Jardim Leonor, São Paulo (SP) – Brasil
**Steering Committee**	Otávio Berwanger (Chair, Co-PI), Henrique Andrade R. Fonseca (Co-PI) Remo Holanda M. Furtado; Hélio Penna Guimarães; Adriano Mendes Caixeta; Pedro Lemos; Marcelo Franken; José Carlos Nicolau
**Predicted duration**	Approximately three years: February 2019 to December 2021.
**Sites**	Approximately 30 sites in Brazil.
**Study Design**	Randomized, prospective, active-controlled, open label, superiority clinical trial, with 1:1 allocation, blind assessment of clinical endpoints.
**Methodological Quality**	Central randomization via web and confidential allocation;<br>blind committee to evaluate and validate diagnostics and endpoints, intention-to-treat analysis.
**Study Objectives**	To evaluate if the increased dose of influenza vaccine in early phase acute coronary syndrome reduces the risk of cardiovascular and respiratory events (death, stroke, AMI, hospitalization for unstable angina, hospitalization for heart failure, emergency coronary revascularization or hospitalization for respiratory infections) compared with late standard dose influenza vaccine.
**Eligibility Criteria**	**Inclusion Criteria:**<br>• Males and females aged ≥ 18 years;<br>• Acute coronary syndrome in-hospital phase.<br>**Exclusion Criteria:**<br>• Participation in another clinical trial;<br>• Refusal to provide consent for the study;

	• Hypersensitivity or anaphylaxis to any component of the vaccine, or Guillain-Barré within up to 6 weeks after previous influenza vaccine;<br>• Have already received the influenza vaccine with the same strains used in the study;<br>• Breastfeeding women;<br>• Pregnant women;<br>• Presenting ACS between the months of December and February;<br>• Hospitalization length for ACS > 7 days.
**Study Intervention**	Inactivated quadrivalent influenza vaccine, with 15 µg hemagglutinin per double-dose viral subtype (total 30 µg, HA), 0.5 mL per dose, IM.<br>Intervention Group: Two simultaneous administrations during hospital phase.<br>Control Group: Single-dose administration 30 days after index event.
**Follow-up**	12 months from inclusion.
**Outcomes**	Outcome consists of cardiovascular and respiratory events (death, AMI, stroke, hospitalization for unstable angina or hospitalization for heart failure, emergency coronary revascularization or hospitalization for respiratory infections).
**Main secondary endpoint**	Outcome consists of major cardiovascular event (cardiovascular death, AMI or stroke)
**Sample Size**	Considering the 12-month incidence of the primary endpoint components in the two study arms and assuming that the double-dose influenza vaccine decreases: Death in 2.0%; AMI in 0.9%; Stroke in 0.3%; Hospitalization for unstable angina in 0.8%; Hospitalization for heart failure in 0.7%; Emergency coronary revascularization in 0.2% and Hospitalizations for respiratory infections in 0.3%; with a sample of 1800 patients, divided into 900 patients per group, one would estimate a *Win Ratio* of 1.41 with a 82.6% power for superiority, considering a 5% significance level.
**Sponsoring**	**PROADI-SUS – NUP:25000.030761/2018-5**

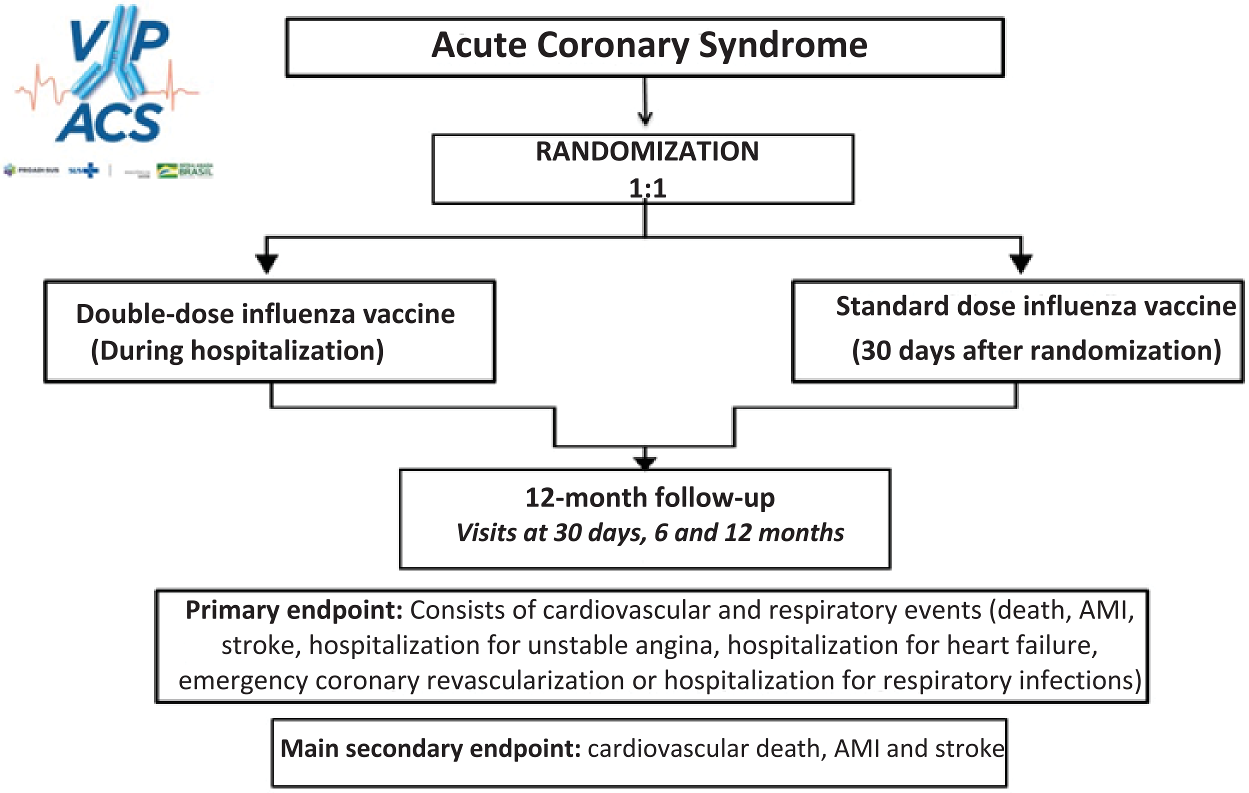

Figure 1. VIP-ACS study flow chart with randomization arms, follow-up and hierarchical endpoints.

CONTENTS

1. INTRODUCTION AND RATIONALE 9
1.1 Rationale for the performance of the study 15
2. OBJECTIVES........ 16
2.1 Primary objective 16
2.2 Secondary objectives........ 16
2.3 Exploratory objectives........ 16
3. STUDY PLANNING........ 17
3.1 Design........ 17
3.2 Eligibility 17
3.2.1. Eligibility Criteria for Hospitalized Subjects........ 17
3.2.2. Eligibility Criteria for Subjects 17
3.3 Randomization Method and Confidential Allocation........ 18
3.4 Masking 18
3.5 Study Procedures 19
3.5.1. Patient management in case of incorrect inclusion or randomization........ 22
3.5.2. Description per visit 22
3.6 Study Interventions........ 25
3.7 Storage and use conditions of the vaccines........ 27
3.8 Safety, concurrent vaccination and immunization campaigns 27
3.8.1. Safety of double use of influenza vaccine........ 27
3.9 HUB-Coordinating site contact – *Helpline*........ 28
3.10 Concurrent therapies 29
3.11 Benefits and risks to patients........ 29
3.12 Lost to follow-up 30
3.13 Sub-study of immunogenicity, biomarkers and virology assessments 30
3.13.1. Collection and storage procedures for biological material........ 31
4.1 ENDPOINTS 31
4.1.1 Primary endpoint 31
4.2 Secondary endpoints........ 32
4.3 Exploratory endpoints........ 32

ALBERT EINSTEIN
SOCIEDADE BENEFICENTE ISRAELITA BRASILEIRA

4.4 Definition of endpoints 33
5. ADVERSE EVENTS 45
6. STATISTICAL ANALYSIS 47
6.1 Sample Size Calculation 47
6.2 Statistical Analysis Plan 49
7. FOLLOW-UP AND DATA CAPTURE SYSTEM 51
7.1 Follow-up 51
7.2 Data Collection System 51
8. ETHICAL ASPECTS AND GOOD CLINICAL PRACTICE 52
8.1 Local Study Approval 52
8.2 Informed Consent Form 53
8.3 Central Study Approval 53
8.4 Study Registration 53
8.5 Data Confidentiality 54
8.6 Reports 54
9. STUDY COORDINATION 54
9.1 Coordinating Site 54
9.2 Steering Committee 54
9.3 Executive Committee 55
9.4 Publications Committee 55
9.5 Adjudication Process 55
9.6 Data Quality Management 56
9.7 Analysis by the Independent Data and Safety Monitoring Committee 57
9.8 Study Sponsor Responsibilities 58
9.9 Responsibilities of Investigators and Sub-investigators of Participating Sites 58
9.10 Monitoring 58
9.11 Publication of Results 59
10. PROTOCOL AMENDMENTS 59
11. REFERENCES 59
12. ANNEX A – Changes in VIP-ACS protocol 64
13. ANNEX B. CONSERVE-SPIRIT[52] 68

ACRONYMS AND ABBREVIATIONS

ACEi – Angiotensin Converting Enzyme inhibitor

ACS – Acute Coronary Syndrome

AE – Adverse Events

AIT – Transient Ischemic Attack

AMI – Acute Myocardial Infarction

AMI – Acute Myocardial Infarction

ANVISA – Brazilian Health Surveillance Agency

CABG – Coronary artery bypass graft

CEC – Clinical Events Committee

CI – Confidence Interval

CRF – Clinical Report Form

CVA – Stroke

CVD – Cardiovascular disease

DD – Double-dose influenza vaccine

EC – Ethics Committee

EDC – Electronic Data Capture

FDA – *Food and Drug Administration*

GBS – Guillain-Barré Syndrome

GCP – *Good Clinical Practice*

GCP – Good Clinical Practices

HA – Hemagglutinin

HAI – *Hemagglutination Inhibition Test*

HD – *Higher dose*

HIAE – Hospital Israelita Albert Einstein

HR – *Hazard ratio*

IAMI trial – *Influenza Vaccination After Myocardial Infarction*

INVESTED trial – *Influenza Vaccine to Effectively Stop Cardio Thoracic Events and Decompensated Heart Failure*

MACE – Major Adverse Cardiovascular Events

OR – *Odds Ratio*

PCI – Percutaneous coronary intervention

RRR – Relative Risk Reduction

SD – Standard dose influenza vaccine

1. INTRODUCTION AND RATIONALE

From systematic reviews of observational evidence (prevalence studies) the World Health Organization (WHO) has provided consistent estimates of causes of death by sex, age, countries and regions. Most recent data show that cardiovascular diseases (CVD) represent the main cause of mortality in both sexes and disability, in Brazil and worldwide. In addition, the accelerated growth of CVD in developing countries represents one of the currently most relevant public health issues. Currently, 80% of deaths by chronic diseases (especially cardiovascular diseases) occur especially in less favored regions of the world[1]; however, better care alternatives, or therapeutic optimization may be a strategy of great value for different social economic conditions.

According to projections from the classic *Murray and Lopez Global Burden of Disease* for the year 2020, there are indications that CVD will not only remain as the main cause of death, but it will also represent the main cause of disability, as the disability adjusted life years (DALY) ascribable to CVD will increase to about 140 to 160 million, which higher proportion will originate from developing countries. This way, in year 2020, the main cause of death and disability in these regions will be CAD and no longer lower respiratory tract infections [2,3].

Among non-traditional cardiovascular risk factors, there is a special interest in the potential association between respiratory tract infections and subsequent cardiovascular events. Observational studies and some minor randomized clinical trials, specifically evaluating influenza vaccine in usual doses versus placebo, showed the decrease of cardiovascular events. A meta-analysis published in 2013 including six randomized clinical trials (RCT) evaluated the effects of the use of influenza vaccine *vs* placebo, or control in patients with high risk of cardiovascular disease, totaling 6,735 patients (mean age of 67 years old, 51.3% female, 36.2% with cardiac history, mean follow-up time 7.9 months). This study showed an association between the vaccine in high cardiovascular risk patients and less subsequent cardiovascular events, with a more marked decrease for those patients after ACS instead of stable coronary disease

(significant interaction). However, there is a discrepancy in these studies related to the findings, and especially methodological limitations limiting ultimate conclusions[4].

Table 1. Summary of randomized clinical trials evaluating influenza vaccine in heart disease patients.

Study	Population	Design	Number of subjects	Baseline characteristics	Follow-up, months (mean)	Region	Number of events	Relative risk of MACE (95% CI)
FLUVACS, 2004	Patients hospitalized with recent ACS or outpatients with stable CAD and planned catheterization	Randomized<br>Blind for the subject and the endpoint evaluator	302	65 years (average)<br>69% male<br>66% acute MI<br>34% stable CAD	12	Argentina	86	0.59 (0.40-0.86)
FLUCAD, 2008	Outpatients with recent ACS or stable CAD and planned catheterization	Randomized<br>Blind for the subject, assisting physician, investigator and endpoint evaluator	658	60 years (average)<br>72.5% male<br>24% catheterization for ACS<br>20% catheterization for unstable angina<br>56% stable CAD	9.8	Poland	46	0.55 (0.30-0.98)
IVCAD, 2009*	Hospitalized patients and outpatients with recent ACS or stable CAD	Randomized<br>Blind for the patient	266	55 years (average)<br>66.2% male	NR	Iran	NR	NR
Phrommintikul et al., 2011	Hospitalized patients with recent ACS	Randomized<br>Open	439	66 years (average)<br>56.3% male<br>47% NSTEMI<br>36% STEMI<br>16% unstable angina	11.8	Thailand	62	0.47 (0.29-0.77)

CAD, coronary artery disease; STEMI, ST-segment elevation myocardial infarction; NSTEMI, non-ST-segment elevation myocardial infarction; CI, confidence interval; MACE, major adverse cardiovascular events; NR, not reported; ACS, acute coronary syndrome. *IVCAD study remains unpublished. In a summary presented in a congress, it was stated that none of the secondary endpoints has been markedly different across the groups in six months; when compared to the occurrence of at least one endpoint for every patient, it is described that the placebo group showed significantly more cardiac adverse events (ACS, coronary revascularization or cardiovascular death), although there is no numerical quantification of this difference.

Another meta-analysis published in 2015[5] found similar observations and reached the same conclusion that there are methodological limitations. Additionally, it must also be considered that the studies performed so far included a small number of patients with clear cardiovascular disease, e primarily patients with coronary disease and/or heart failure. Also, small observational studies showed a potential protection factor of the influenza vaccine against cerebrovascular accident (CVA), but due to limitations

inherent to the observational studies[6] there is no pragmatic evidence that this vaccine may also decrease this type of vascular event.

Pilot trials in heart failure, a condition that leads to a decreased seroconversion rate to vaccination, showed that increasing influenza vaccine dose to 30 µg corresponded to an increase in antibodies titer, increasing protection against influenza[7,8]. A major fact is that few adverse effects were seen when doubling the dose of the vaccine, most of them only at the administration sites.

Upon increasing the dose for a higher seroconversion, recent studies have revealed that the high dose vaccine with 60 µg in patients above 65 years old considerably increases immunogenicity against influenza epitopes, reflecting a higher efficacy in reducing respiratory tract infections and also hospitalizations[9-11]. This increase in elderly population protection may be a prevalent factor in the decrease of hospitalizations and death for different causes, especially those with background cardiovascular disease. In a way that it could be postulated that the increased dose of influenza vaccine (60 µg) could show a potential do reduce cardiovascular events, higher when compared to the standard dose of 15 µg, in this population[12].

In this sense, an initiative led by *Brigham and Women's Hospital* investigators and financially supported by the US National Institute of Health (NIH), *INVESTED* study[13], evaluated the effect of the influenza vaccines in increased doses in patients with recent hospitalization for heart failure (last 2 years) or acute myocardial infarction (last year). Based on the above-mentioned idea, this study tested the additional benefits of the use of the 60 µg dose in reducing cardiovascular events in outpatients. As a limitation, it does not propose to test the vaccine during hospitalization, at the most critical moment of the heart disease patient in terms of risk and compliance to the proposed measures. Interestingly, the vaccines used in this trial correspond to an increased trivalent dose versus standard quadrivalent dose vaccine, in a way that the findings could be related to the composition of the vaccine, the vaccine strains or concentration of certain strains in cardiovascular protection[14-18], opening a large window to discussion. So that the

recently published results revealed that the study was interrupted early for futility, as after the inclusion of 5260 patients, of the 9200 planned, the DSMB instructed for the interruption as there was no benefit of the intervention with an increased dose of trivalent vaccine (HR 1.06; [95% CI: 0.97-1.17]) in decreasing mortality or cardiopulmonary hospitalization, compared to the quadrivalent standard dose[49].

The hypothesis of decreasing the chances of an infection during or after an AMI has been tested by IAMI study, in Scandinavia. This trial evaluated the intra-hospital use of a usual dose of quadrivalent influenza vaccine of 15 µg *vs* placebo in patients with AMI eligible to percutaneous coronary intervention[19]. The IAMI trial tested the hypothesis of the benefit of influenza vaccine in decreasing CVD events in 12 months, vaccinating the patients during the period of higher immune activation, i.e., within the 72-hour window after the angiography. The results were promising in the context of patients after an AMI, as the vaccinated group showed a 28% decrease (HR 0.72; [95% CI: 0.52-0.99]) of the composite primary endpoint (total death, AMI or stent thrombosis) during follow-up[50]. It is highlighted that this study was interrupted early due to the COVID-19 pandemic in 2020 with a total of 2532 patients included, as the prediction was a total of 4400.

One of the main issues about the applicability of the INVESTED study was the exclusion of those patients with indication of influenza vaccine, as there is a high priority recommendation for the vaccine in chronic heart disease patients[20] and for those above 50 years-old[21], it may be inferred that, either the study population will not be representative of all post-ASC patients, as most of them will have a priority vaccine indication, or a number of patients with indication for routine influenza vaccine will not be vaccinated for being part of the control group, limiting the comparison.

Also, importantly, INVESTED and IAMI trials randomizations were only performed during viral circulation periods in the northern hemisphere, i.e., only during winter. This approach may be justified due to the different findings pointing to a higher risk of cardiovascular events during this season, presenting well defined epidemic periods[22,23] as suggested in figure 2.

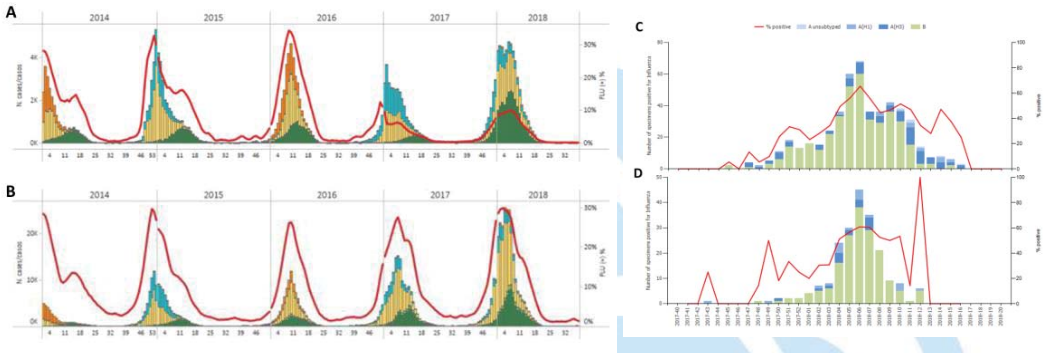

Figure 2. Representation of the seasonality of the influenza virus in the northern hemisphere. Figures A and B show the changes from the year 2014 to week 32 of 2018. Figures C and D represent the seasonality of the influenza virus during the 2017/18 season in Sweden and Denmark, respectively. The colors of the columns represent the subtypes of influenzas virus (adapted from PAHO/WHO, 2018; ECDC/EISN, 2018).

However, in addition to the limitations inherent to each study design, the results of the above mentioned ongoing studies could not be entirely applied to Brazil, as the country shows a consistent viral circulation during all the months of the year and different across the regions, and the reasons for this are not clear[24,25]. Additionally, the inversion of seasonality of certain subtypes of influenza virus in Brazil during the same year is still common[26] (figure 3), and in case the virulence has an important role in the risk for CV events, other measures and immunization criteria must be adopted, in addition to those already described during traditional annual immunization campaigns.

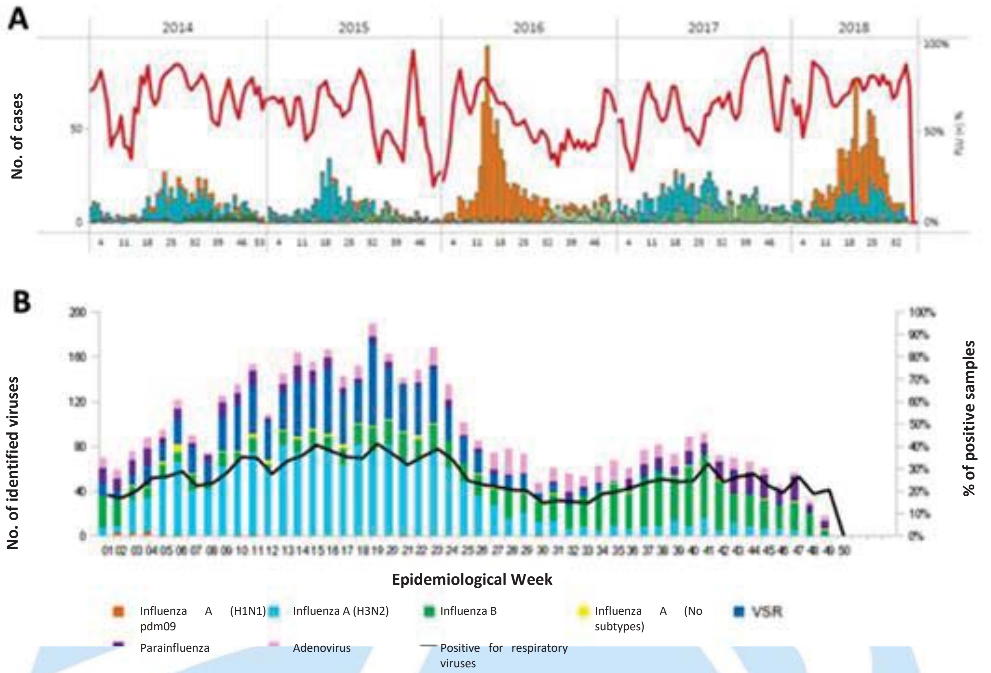

Figure 3. Representation of the seasonality of influenza virus in Brazil. Figure A shows seasonal changes from year 2014 to week 32 of 2018. Figure B shows seasonality of the influenza virus throughout the year 2018 (adapted from PAHO/WHO, 2018; SVS/MS-Brasil, 2017).

The seasonality condition of influenza in Brazil opens a large window of possibilities between infection and cardiovascular disease, in a way that the vaccination throughout the year, or during most part of it, may be a cardiovascular protection and economic gain tool, compared to countries with a well-defined seasonality. For such idea, a survey recently performed in South Korea showed that standard dose (15 µg) trivalent vaccine against influenza could reduce the costs with the management of coronary syndrome in up to 86 million dollars/year[27]. However, additional benefits with HD vaccines for this purpose are still to be studied.

This high dose vaccination approach shows to be safe and have a seroprotection superior to usual dose vaccines, even in those subjects with some immunosuppresion and/or immunosenescence condition[28,29], very familiar situations to patients who have some background cardiovascular disease, such as heart failure, chronic renal disease, diabetes and elderly.

Brazil annually promotes immunization campaigns for the population at higher risk for adverse effects due to influenza infection, through conventional dose trivalent vaccine, manufactured in the country by Instituto Butantã, under SANOFI-Pasteur-France license[30]. Even with millions of doses distributed to primary care, a continuous decrease in the population search for the vaccine has been seen, even in those patients at higher risk[31-33].

Vaccination with an increased dose throughout the year could be a cost-effective intervention of great value to decrease cardiovascular events, as this is an easy compliance, administration and low-cost approach, when compared to continuous use medication. Additionally, vaccination during hospitalization is an opportunity, as this is the only moment that the complete compliance with the proposed treatment is practically guaranteed. However, there is no pragmatic evidence that could support this idealization.

1.1 Rationale for the execution of the study

Cardiovascular disease is a great burden in the context of public health, as well as the low pharmacological compliance of patients with non-communicable chronic diseases. However, we have no vaccine efficiency data to reduce cardiovascular events in acute phase, and few studies have evaluated the cardioprotective potential of the influenza vaccine were conducted in countries with well-defined seasonality and/or vaccine doses not licensed in Brazil. Therefore, a study evaluating the HD vaccine in a period covering all influenza virus seasonality in Brazil can bring important findings to the different scientific gaps, as well as to clarify issues on the potential benefit of double-dose vaccine, with no contraindications, immediately after an atherothrombotic event. If a real benefit is shown, it may also be a future therapeutic tool adjuvant to traditional drug therapy for the prevention of cardiovascular events.

2. OBJECTIVES

2.1 Primary objective

To evaluate if increased doses of influenza vaccine at hospital phase, when compared to outpatient vaccine in the usual dose, decrease the risk of combined cardiorespiratory events (death, AMI, stroke, hospitalization for unstable angina, hospitalization for heart failure, emergency coronary revascularization or hospitalization for respiratory infection) in patients with acute coronary syndrome.

2.2 Secondary objectives

The main secondary objectives aim to evaluate whether, in patients during the acute phase of an ACS, the increased dose influenza vaccine is superior to the late vaccine to prevent the following endpoints: AMI; CVA; TIA; cardiovascular death; total mortality; pulmonary death; hospitalization for unstable angina; need of coronary revascularization (emergency and non-emergency); acute myocardial injury; hospitalization for heart failure; hospitalization for upper airways and pulmonary infection, and stent thrombosis.

2.3 Exploratory objectives

To determine the rate of adverse events related to double-dose vaccine; to compare the seroprotection between the two treatment arms with the occurrence of endpoints; occurrence of primary endpoint in up to 45 days after randomization.

3. STUDY PLANNING

3.1 Design

Randomized, active-controlled, multicenter, open label, superiority, parallel groups and 1:1 allocation trial with blind evaluation of clinical endpoints and intention-to-treat analysis.

3.2 Eligibility

3.2.1. Eligibility Criteria for Hospitalized Subjects

Major Brazilian hospitals, both public and private (but preferably public hospitals, with outpatient care for patients at high cardiovascular risk), connected to a Basic Health Units reference network, will be included.

3.2.2. Eligibility Criteria for Subjects

3.2.2.1 Inclusion Criteria

- Males and females aged ≥ 18 years;
- Patients with acute coronary syndrome.

3.2.2.2 Exclusion Criteria

- Participation in another clinical trial.
- Refusal to provide consent for the study;
- Hypersensitivity or anaphylaxis to any component of the vaccines;

- History of Guillain-Barré Syndrome 6 weeks after an influenza vaccine, previously to study inclusion;
- Have already received the influenza vaccine with the same strains used in the study (comprises trivalent or quadrivalent vaccines with the same strains of the study);
- Pregnant women;
- Breastfeeding women;
- Presenting ACS between the months of December and February;
- Hospitalization length for ACS > 7 days.

3.3 Randomization Method and Confidential Allocation

The patients' randomization list will be generated considering a random function with equal allocation probability to one of the vaccine therapy groups and in relation to each investigational site. Each study vaccine group will be numbered and only the numbers will be used in the randomization, which will be performed through a randomization central electronic system of the project coordinating site at HIAE, guaranteeing the secrecy of the allocation. In special cases, where randomization problems occur, the coordinating site will contact the investigational site with the actions to be taken.

3.4 Masking

This is an open-label study, where treatment allocation will be open to investigators, medical care team and patients. Clinical endpoints will be evaluated and validated by a Blind Independent Committee, where the blinding will be interrupted at

the end of the inclusion of the last patient, or for safety conditions related to the use of influenza vaccines, or for extraordinary necessity (case of an influenza pandemic) requiring changes in vaccines and their compositions.

3.5 Study Procedures

After the ethics approval of the participating sites, each site will receive, and initiation visit for the training and qualification of the research team in the study procedures.

The study will start randomizations as soon as 2009 influenza vaccines are available (February/2019) and will continue until November/2020, even after the national influenza vaccine campaign for the mentioned years. Remembering that between the months of February/March 2020 a new randomization period is started, with the availability of influenza vaccine for the due year. In such a way that the project will have two patient inclusion periods:

Period 1 – Influenza vaccine 2019 – Between the months of February and November 2019;

Period 2 – Influenza vaccine 2020 – Between the months of February and November 2020.

Period 3 – Influenza vaccine 2021 – Between the months of February and November 2021.

There is a possibility of the inclusion of another vaccination period, if the steering committee points a need of a higher patient inclusion depending on the number of events related to the primary endpoint.

Patients will be randomized after the signing the ICF in the specialized site (HUB) where they were cared for. Inclusion and exclusion criteria will be evaluated after the evaluation of the acute coronary syndrome, confirmed by electrocardiogram and/or cardiac enzyme tests**.** Randomization may be performed in up to seven days from the initial hospitalization related to the index event, ACS.

The indication or not of percutaneous coronary intervention will not prevent entering the study, with attention to the description of the vaccination in the CRF, both before or after the procedure.

As the beneficial effects of influenza vaccine seem to be connected to its administration in the **early ACS period**, the sites are recommended to give it **as soon as possible at the onset of symptoms**, but with attention to each unit and each case priorities of care. The failure to comply with this recommendation in no way prevents the inclusion of the patient, as he/she may also receive the vaccine at a time close to the hospital discharge.

Vaccine during the hospitalization for the index event will be given in two doses, divided into 0.5 mL intramuscularly in the deltoid, sequentially in each of the patient's arms; in case the vaccine cannot be given in the upper limbs, there is a possibility of giving the vaccine in the lower limbs by employing the vaccination strategy in each member.

Patients randomized to the late standard dose influenza vaccine group (0.5 mL) will receive this vaccine at the 30 days ±5 visit (V1) after randomization. For every patient who is still hospitalized at V1, the visit and the vaccine will take place in a hospital setting.

After the vaccine all the patients will be evaluated on day **7(+2) and on day 28(+3)**, by interview or phone, for the evaluation of potential local and systemic adverse occurrences that might occur, i.e., to evaluate potential reactogenic/adverse effects. In case of some adverse reaction, it will be controlled by therapies for improvement, control of signs and symptoms, and subsequently described in the eCRF.

Pharmacological therapies for the treatment of ACS will be those provided for by current guidelines, as well as by the protocols standardized by each research site.

Patients must return to the proper investigators sites for follow-up visits in 30 days ±5 days (V1), 06 months ±10 days (V2) and 12 months ± 20 days (V3) as of the randomization date. In special cases, exceptionally, remote contact may occur for the 6

(V2) and 12 (V3) months visits. During this period, patients will also be contacted by the research teams by phone to keep the compliance with the study and to monitor potential clinical events, as described in figure 1.

Understanding the study design, the patients will be randomized in up to seven calendar days of the index event (ACS). In the randomization defines that the patient will receive the double-dose, it must be given during the hospitalization phase for this event (at any time, during hospitalization). Patients randomized to late administration of standard dose influenza vaccine will receive a single dose at the 30 ± 5 days visit from randomization. The other follow-up visits will be at 06 months ±10 days and 12 months ±20 days from randomization. All the patients will have three visits established in the study, as in **figure 4**.

Double-dose vaccine at the hospitalization for index event and *Late administration of standard dose vaccine at the 30-day visit after randomization*:

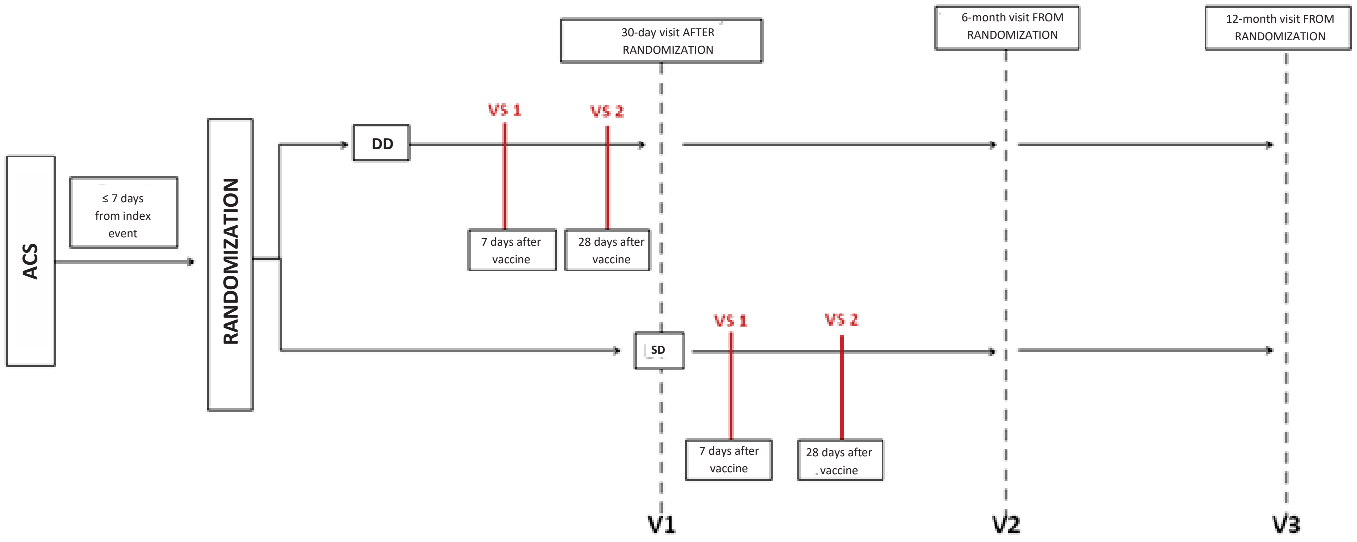

Figure 4. *Description of study follow-up visits. Follow-up visits are described as V1, V2, V3. Safety visits are described in red.*

Keys: DD = Double-dose vaccine; SD = Standard dose vaccine; SV = Safety Visit.

3.5.1. Patient management in case of incorrect inclusion or randomization

In cases where the inclusion of the ACS patient is inadequate (for lack of criterion or diagnosis error or inversion of groups at randomization) or in case of error in the administration of the vaccine, the investigational HUB must contact the coordinating site that will evaluate the continuation, discontinuation or adequacy of the randomization of the patient involved. All the decisions made on this matter must be documented and described together with the patient's information in the CRF.

3.5.2. Description per visit

The procedures intended for each visit will be performed according to the description below:

Table 2. Description of the procedures and visits of the VIP-ACS study

	**Hospital phase**	**7 days from vaccination**<br>***Safety***	**28 days from vaccination**<br>***Safety***	**Hospital discharge**	**30 days**<br>**V1**	**6 months from Inclusion**<br>**V2**	**12 months from Inclusion**<br>**V3**
Evaluations							
Screening/Randomization	X						
Review of eligibility criteria	X						
Previous medical history	X						
Socio-demographic data	X						
Medications in use	X			X	X		
ICF	X						
Clinical Events Evaluation		X	X	X	X	X	X
**Physical Measures**							

Blood pressure, pulse and vital data	X						
Physical examination	X						
Anthropometry	X						
**Study intervention**							
Vaccination	X				X		

- ***ADMISSION – HOSPITAL PHASE - RANDOMIZATION***

The data collection team must check the following patient's data:

- Inclusion/exclusion criteria;
- Demographics;
- Clinical medical history;
- Vital signs;
- Revascularization tests or interventions, when indicated;
- Tests related to hospitalization;
- Randomization

If the patient is randomized to double dose vaccine, it will be given during the hospitalization for the index event.

Patients randomized to late standard dose influenza vaccine will receive it at the visit 30 days after randomization.

SV1 **- Safety evaluation after vaccine (7 /+ 2 days)**

-Evaluation of adverse events 7 days after the administration of double-dose vaccine (in person or by phone).

SV2 **- Safety evaluation after vaccine (28 /+ 3 days)**

-Evaluation of adverse events 28 days (+ 3 days) after the administration of double-dose vaccine (in person or by phone).

- **HOSPITAL DISCHARGE**

At discharge, describe:

- Clinical Evolution during hospitalization;

- Medications prescribed;

- Occurrence of endpoints.

- **V1 - 30 DAYS ± 5 DAYS FOLLOW-UP VISIT**

Visit 30 days after randomization This visit will be calculated from the date of randomization.

Patients randomized to the late standard dose of influenza vaccine will receive it at this visit.

At this visit, patients who were given double-dose vaccine will be evaluated regarding the clinical evolution, endpoints of interest to the study and adverse events:

- Clinical Evolution;

- Occurrence of endpoints.

SV1 **- Safety evaluation after vaccine (7 /+ 2 days)**

- Evaluation of adverse events 7 days after the administration of standard dose vaccine (in person or by phone).

SV2 **- Safety evaluation after vaccine (28 /+ 3 days)**

- Evaluation of adverse events 28 days (+ 3 days) after the administration of standard dose vaccine (in person or by phone).

- **V2 - 06 MONTHS ± 10 DAYS FOLLOW-UP VISIT**

- Clinical Evolution;
- Occurrence of endpoints;
- Occurrence of adverse events;

- **V3 - 12 MONTHS ± 20 DAYS FOLLOW-UP VISIT**

- Clinical Evolution:
- Endpoints;
- Adverse events;
- Study closure

3.6 Study Interventions

Patients will be randomized and assigned in a 1:1 ratio to each one of two study arms: double-dose influenza vaccine during hospitalization for the index event or late standard dose vaccine at the 1-month follow-up visit. Once assigned to one of the vaccine groups, the care of all the patients into that study arm will follow the same procedure.

After the randomization procedure, the patient will be guided to receive:

- ✓ Quadrivalent, fragmented and inactivated, double-dose influenza vaccine (30 μg) during hospitalization;

Or,

- ✓ Quadrivalent, fragmented and inactivated, standard dose influenza vaccine (15 μg) at 30 days.

3.6.1 Study vaccine

The vaccine used in the study will be similar to the national influenza vaccine campaign (trivalent) of the year 2019, 2020 and 2021, however with the addition of another strain B (quadrivalent). This way, using the same vaccine used nationally containing the same hemagglutinin antigens recommended by the World Health Organization for the year mentioned.

In the year 2019, the fragmented and inactivated seasonal influenza vaccine consisting of 15 µg/mL of hemagglutinins from *Myxovirus influenzae* virus strains propagated in embryonated chicken eggs *[(A/Michigan/45/2015 (H1N1) pdm09-like virus; A/Switzerland8060/2017 (H3N2)-like virus; B/Colorado/06/2017-like virus (B/Victoria/2/i87 linage); Phuket/3073/2013 (B/Yamagata/16/88 lineage)*]] in a single-dose suspension for injection[34], (Fluarix® Tetra quadrivalent, fragmented and inactivated influenza vaccine, *GlaxoSmithKline Biologicals NL der SmithKline Beechman Pharma GmbH & Co*). While for the year 2020, the vaccine recommended by the Brazilian Ministry of Health, with its formulation strains endorsed by WHO to the southern hemisphere containing the following strains: an A/Brisbane/02/2018 (H1N1)pdm09-like virus; A/South Australia/34/2019 (H3N2)-like virus; B/Washington/02/2019-like (B/Victoria lineage) virus; B/Phuket/3073/2013-like (B/Yamagata lineage) virus(Fluarix® Tetra quadrivalent, fragmented and inactivated

influenza vaccine, *GlaxoSmithKline Biologicals NL der SmithKline Beechman Pharma GmbH & Co*)[35].

If the study requires another wave of inclusion, the study will use a fragmented and inactivated, quadrivalent vaccine against seasonal influenza, consisting of 15 µg/mL of hemagglutinins from *Myxovirus influenzae* virus strains propagated in embryonated chicken eggs from the same manufacturer mentioned above, with antigens in its composition recommended by the Brazilian Ministry of Health and the World Health Organization for the southern hemisphere of that year of inclusion.

3.7 Storage and use conditions of the vaccines

For the appropriate storage of the vaccines, the HUB will use a vaccine refrigerator with temperature log that must fluctuate between +2 and +8°C. Daily records will be made, in three shifts, on the temperatures indicated in the refrigerator display. In case of more than two measurements out of the above-mentioned temperature range, the person in charge at the HUB must take measure to check for the reason of the fluctuation and make the adjustments based on the study operations manual. If the problem persists, the HUB must contact the coordinating site about the occurrence, and take measures for changes or adjustments. In case of more than 24 hours of disturbance in the target temperature, the coordinating center must be contacted, and measures taken to dispose and change the vaccines.

3.8 Safety, concurrent vaccination and immunization campaigns

3.8.1. Safety of double use of influenza vaccine

Based on previous studies, in healthy populations and with different immunosuppressive conditions, it is expected that the double dose of influenza vaccine

will present higher cases of local adverse reactions (AR), without compromising the patient's health[36-38]. An influenza vaccine that is currently approved by the FDA and ANVISA[39,40] has 4x the usual dosage, without significantly increasing the AE[41].

3.8.2 Concurrent use of other vaccines

In case the patient uses any other vaccine during the study follow-up (12 months), the description of this vaccine **must be described in the eCRF**, as an example of vaccines: Measles, Yellow Fever, Hepatitis B, Triple Viral, among others.

3.8.3 Vaccination during national vaccination campaigns

During the 12 months following randomization, patients will be **instructed not to receive any influenza vaccine**, even if national influenza vaccine is available. The patient will receive their next influenza vaccine after the 12-month period of randomization, under the guidance of the investigating site. In special cases of influenza local outbreaks or epidemics, the coordinating site will instruct the investigating sites on the appropriate measures to vaccinate the participants.

3.9 HUB-Coordinating site contact – *Helpline*

The study will have a telephone helpline between the HUB and the investigating site for any urgent or emergency questions related to patient issues, such as randomization, vaccines, inclusions and/or unexpected adverse events.

3.10 Concurrent therapies

The VIP-ACS study will not provide formal guidance on the predetermined use of therapies concurrently with the influenza vaccine. It will be advised that all HUB perform the usual care at each unit, observing evidence-based patient management, through current recommendations and guidelines for ACS and other conditions, inherent to the study and/or preexisting inclusion event. HUB health teams will be instructed to recommend the patient, at hospital discharge, to follow healthy lifestyle habits, including the practice of regular physical exercise, balanced eating habits and pharmacological compliance with prescribed drugs.

3.11 Benefits and risks to patients

Based on current recommendations for the use of influenza vaccines, patients in secondary prevention are those categorized as priority groups for vaccination during annual influenza immunization campaigns. These recommendations are due to the high risk of infection-derived events in this population. Thus, the VIP-ACS patients will be randomized during the hospital phase, that is, they received the vaccine soon after the CV event or within 30 days after discharge, and the patient does not need to look for the places where the vaccination is performed during campaigns. Another important point is regarding the vaccination of the VIP-ACS project to take place during the national immunization campaign and after its closing (until November), covering the immunization of many individuals who would not receive the vaccine, and now belong to the priority group, and who would be without this vaccine coverage, even with the circulation of the influenza virus after the end of the campaign.

The main risks are related to adverse events of the influenza vaccine, which may be systemic or local, depending on age, risk factors for infections, double use of the vaccine, among others. The possibility that these AEs will occur will be reported to patients, as well as their therapeutic and prophylactic means. The occurrences of these AEs will be described in the CRF and managed by the HUB medical teams.

3.12 Lost to follow-up

Patients who do not attend the follow-up visits or do not return phone contacts and/or emails, and did not describe their own willingness to discontinue their participation in the study, will be actively searched through family members and close personal contacts, described in the inclusion documents and in the ICF of VIP-ACS study. These patients will be instructed to continue in the study and efforts will be made by the team of HUB investigators for the maintenance of this patient. Cases of voluntary discontinuation will be described in the CRF, and the patient will be instructed to continue their follow-up at their Hub or referral site.

In cases of pregnancy confirmation during the study follow-up, the results obtained from this/these patient(s) will be reported in the CRF and will not be excluded from analyses and visits during the study follow-up.

3.13 Sub-study of immunogenicity, biomarkers and virology assessments

The VIP-ACS study will select sites in different regions of viral seasonality to assess seroconversion to vaccination, through serological assays measuring the changes between geometric means of antibody titers against hemagglutinins present in the vaccine. This assessment allows us to quantify the seroconversion, which is considered ideal, when antibody titers are elevated by 4 times after vaccination[42]. Elderly patients (>65 years-old) have a lower conversion rate when compared to younger individuals[43],

and possibly the increase in seroconversion due to the use of an increased dose of vaccine may have implications for the primary endpoints of the study. Additionally, immune responses after vaccination, through cytokines or other biomarkers may also be linked to the findings. Another important point is the viral typing of the patients included in the study, since certain viral subtypes may be more immunogenic, and thus be related to the type of endpoint, as already speculated by previous studies[43-47].

For these analyses, 1000 patients will be randomly invited from different sites, among those who will participate in the VIP-ACS study. Prior to the study vaccines, during the hospital phase, patients will be asked to perform a venipuncture for blood collection. Another blood collection will be performed in the 30 (+/-5) days outpatient visit, in the 6 months (+/-10 days) and 12 months (+/- 20) days visits.

The material derived from the blood collection will be stored under ideal conditions for future analysis of seroconversion, plasma and viral biomarkers, and subsequent analysis with the endpoints found.

3.13.1. Collection and storage procedures for biological material

Samples will be collected from the participants and during their visits processed in serum and plasma and stored at -70˚C for the aforementioned evaluations. The methods and tests applied for material processing, analysis and methods will be described in a laboratory operation manual.

4.1 ENDPOINTS

4.1.1 Primary endpoint

Hierarchical combined endpoint of death, acute myocardial infarction, stroke, hospitalization for unstable angina, hospitalization for heart failure, emergency coronary revascularization or hospitalization for respiratory infections.

4.2 Secondary endpoints

The study will have the *main secondary* endpoint which will consist of major cardiovascular events (cardiovascular death, AMI or stroke).

Other secondary endpoints include time to first event of the following endpoints:

- Individual components of the primary endpoint;
- Total mortality;
- Hospitalization for unstable angina;
- Hospitalization for respiratory infections;
- Need for coronary revascularization due to ischemia;
- Transient ischemic attack (TIA);
- Acute myocardial injury; types of infarction according to the 4th Universal Definition[48]
- Definite or probable stent thrombosis;
- Hospitalization for SARS-Cov2 virus infection (COVID-19).

4.3 Exploratory endpoints

Occurrence of local and systemic adverse events at 7 and 28 days after vaccination. Occurrence of serious adverse events related to vaccination and serious adverse events within 12 months; Occurrence of the primary endpoint between treatment groups within 45 days of randomization; Vaccine antigen responses ≥ 4 times baseline by hemagglutination inhibition test.

4.4 Definition of endpoints

4.4.1 Cerebrovascular Accident (CVA);

Defined as an episode of acute focal or global neurological deficit caused by vascular damage to the brain as a result of hemorrhage or infarction. Vascular damage must be documented by neuroimaging OR the deficit must persist for at least 24 hours. Stroke is subdivided into ischemic, hemorrhagic or undetermined.

Neuroimaging scan reports must be submitted by the site. Preference will be given to magnetic resonance scans for diagnosing ischemic stroke, but if these are not available, computed tomography (CT) scans will be accepted. Cases of incidental findings on imaging scans (such as micro-hemorrhages, silent infarcts, etc.) without corresponding neurological manifestation will not be positively adjudicated as a vascular event.

Cases evolving to death within 24 hours and before the imaging scan can be obtained, when it is believed that the death occurred as a direct consequence of the acute vascular insult, will be positively adjudicated as stroke.

4.4.1.1 Ischemic stroke

Defined when vascular damage occurs as a consequence of central nervous system tissue infarction, usually caused by atherothrombosis, embolism or low flow. In cases of hemorrhagic transformation of ischemic stroke, the event will still be categorized as ischemic. The exam of choice is the imaging scan (CT or MRI) showing an infarction in an area compatible with the clinical deficit. However, cases of stroke with symptoms <24 hours in duration where CT is normal will be categorized as ischemic if other imaging scans are not available during the index event and if the deficit has persisted for more than 24 hours or the patient evolved to death as a result of the event.

4.4.1.2 Hemorrhagic stroke

Defined when vascular damage occurs as a result of primary central nervous system

hemorrhage. Ischemic stroke with hemorrhagic transformation does not fit this definition. Bleeding can be intraparenchymal, intraventricular or subarachnoid and must be non-traumatic in nature. Cases of subdural hematomas will not be considered as hemorrhagic stroke.

4.4.1.3 Undetermined stroke

When the available data are insufficient to categorize the event as ischemic or hemorrhagic.

4.4.2 Transient Ischemic Attack (TIA)

Defined as an acute episode of focal neurological deficit caused by ischemia of the brain, spinal cord or retina, when there is no evidence of infarction on neuroimaging scan and the deficit has persisted for < 24 hours.

4.4.3 Acute Myocardial Infarction

Universal definition of infarction (according to the 4th Universal Definition)[48]

Universal definition of infarction

Myocardial infarction is defined as evidence of myocardial necrosis (usually through increased biomarkers) associated with evidence of ischemia (through ECG, symptoms, or evidence on imaging scan). Therefore, the diagnosis of MI necessarily requires the following two criteria:

a) Elevation of myocardial necrosis markers (troponin, CKMB or CPK), with a rising and falling pattern, and at least one value above the 99th percentile for the reference value; AND

b) Clear evidence of myocardial ischemia, manifested as one of the following findings: ischemic symptoms (e.g.: precordial pain); new or presumably new ST-segment or T-wave ECG changes or new left bundle branch blockade; emergence of a new Q wave on the ECG; emergence of new segmental motility or perfusion abnormalities on

imaging scan; identification of intracoronary thrombus on angiography or autopsy.

The 4th Universal Definition of MI (4UDMI) also created the category of non-ischemic myocardial injury, in cases where there is an elevation of biomarkers (item a above), but without clear evidence of ischemia by the context of the exams and clinical condition. Examples of situations associated with a potential etiology of non-ischemic myocardial injury are sepsis, decompensated HF, trauma, post-cardiac procedures (except cases that are diagnosed as periprocedural MI – see below).

Preferably, the biomarker of choice for diagnosing MI will be cardiac troponin. If this is not available, the dosage of CKMB or myoglobin can be used as an alternative. Other biomarkers (e.g.: transaminases, lactate dehydrogenase, total CPK) will not be considered for the diagnosis of MI by this committee.

Event reports must be submitted by the sites along with lab tests and ECG tracings obtained during the index event. If these are not available, reviewers may consider the medical report or description of the tracing obtained from the medical admission reports or discharge summaries.

4.4.3.1 Types of infarction

All events positively adjudicated as an MI will be sub-ranked according to the following criteria, based on the best assessment available from the submitted source documents.

4.4.3.2.2 About the 4DUIM classification

a) Type 1 infarction (Spontaneous myocardial infarction): Are those MIs secondary to rupture, erosion, dissection, fissure or erosion of an unstable atherosclerotic plaque, resulting in a partially or totally occlusive luminal thrombus. The patient may have underlying CAD or not, or even have non-obstructive CAD.

b) Type 2 infarction (Infarction secondary to ischemia due to supply/demand imbalance): They are secondary to a condition other than CAD, leading to reduced supply or increased myocardial demand, for example, vasospasm, coronary embolism, endothelial dysfunction, severe anemia, brady or tachyarrhythmias, hypotension,

respiratory failure or hypertensive crisis. Situations where there is an increase in biomarkers associated with one of these causes (e.g.: bleeding with secondary anemia) but without clear evidence of ischemia (symptoms, ECG, or imaging scan) will be classified as non-ischemic myocardial injury.

c) Type 3 infarction (Infarction resulting in death before biomarkers are available): Cardiac death with symptoms suggesting myocardial ischemia and new ischemic changes on the ECG (e.g., ST changes or new LBBB), with death occurring before blood samples can be obtained for the measurement of biomarkers, or before the time in which these can rise.

d) Type 4 infarction (Related to PCI)

d.1 Type 4a: Periprocedural MI - defined when there is elevation of biomarkers > 5 X ULN within the first 48 hours after PCI associated with at least one of the following findings: symptoms suggesting ischemia; new ischemic changes in the ECG; angiographic evidence of loss of a branch or reduction in coronary flow; imaging scan showing loss of previously viable myocardium.

d.2 Type 4b: MI related to a stent thrombosis: MI related to stent thrombosis confirmed by angiography or autopsy

d.3 Type 4c: MI related to stent restenosis: MI related to restenosis (lesion > 50% obstruction) in a site previously treated with PCI.

e) Type 5 infarction: MI related to CABG surgery. defined when there is elevation of biomarkers > 10 X ULN within the first 48 hours after CABG surgery associated with at least one of the following findings: symptoms suggesting ischemia; new ischemic changes in the ECG; angiographic evidence of graft or native vessel occlusion; imaging scan showing loss of previously viable myocardium.

4.4.3.2.3 About the ECG

a) ST-segment elevation myocardial infarction (STEMI): MI with ECG during index event showing persistent ST-segment elevation ≥ 1 mm in two or more contiguous leads (≥ 2 mm if leads V2-V4) or new or presumably new LBBB.

b) Non-ST elevation myocardial infarction (NSTEMI): NI with ECG not showing either

of the two changes mentioned above.

c) Myocardial infarction with indeterminate ECG: Cases where the index event ECG is unavailable or was not performed.

4.4.3.2.4. About the biomarkers peak

Biomarkers dosages (troponin or CKMB) will be requested from local labs, together with the normal reference values. The event adjudication committee's database will then store this data and the MIs will be classified according to the size estimated by the biomarkers peak in number of times of the ULN.

4.4.3.5. Non-Ischemic Myocardial Injury

Cases that meet the criteria for biomarkers for MI, but where there is no evidence of ischemia based on ECG or imaging scans or symptoms will be classified as non-ischemic acute myocardial injury.

4.4.3.6. Silent infarction

Defined as the appearance of a new Q wave or new area of non-viable myocardium on imaging scan (e.g.: echocardiogram) or anatomopathological finding of infarction, without a documented clinical condition or documented change in biomarkers.

4.4.4 Deaths (Mortality)

All deaths will be reviewed by the independent adjudication committee and the main cause leading to the fatal outcome will be determined. Source documents related to medical evolutions, test results, discharge summaries and death certificates will be requested from the sites when submitting the event.

4.4.4.1. Cardiovascular (CV) Death

It includes death from coronary heart disease, sudden cardiac death, death from heart failure (HF), death from stroke, death from a CV procedure, death from CV hemorrhage, and death from other CV causes.

I. *Death from coronary heart disease*

Any death with a clear relationship to underlying coronary disease, such as death from myocardial infarction (MI), sudden cardiac death, and death as a complication of a coronary revascularization procedure.

Death caused by MI will be defined as death caused by a cardiovascular mechanism, as a direct consequence of MI (e.g., arrhythmia, cardiogenic shock, HF, mechanical complications, etc.), and occurring within 30 days of MI. Deaths caused by a procedure used to treat a heart attack (e.g.: coronary artery bypass graft surgery or coronary angioplasty) should also be considered as death due to MI. Sudden death cases preceded by a case positively adjudicated as IM will also count as deaths due to MI. This includes cases with MI discovered during necropsy, or when the diagnosis is made before the possibility of timely collection of markers (see definition of MI type 3 below).

II. *Sudden Cardiac Death*

It refers to unexpected death, not directly consequential to an MI, and includes the following possibilities:

a) Witnessed death occurring without new symptoms or after worsening of old symptoms;

b) Witnessed death, occurring within the first 60 minutes after the onset of new symptoms, unless the symptoms suggest an acute infarction (see definition of death from MI and definition of MI);

c) Witnessed death, attributed to a documented arrhythmia (e.g.: in ECG tracing or while reading an implanted cardiac device, such as pacemakers and cardio defibrillators);

d) Death within 30 days of failed CPR due to cardiac arrest of undetermined cause;

e) Unexpected death, of unknown and unwitnessed cause in an individual known to be well in the previous 24 hours and with no clear signs that it may have been due to a non-cardiovascular cause (e.g.: trauma, poisoning. If the patient's status is not known in the 24 hours preceding the event, it should be considered as death of undetermined cause.

III. *Death caused by heart failure (HF)*

It refers to death that occurs in the context of worsening HF signs/symptoms, such as cardiogenic shock, pulmonary edema or arrhythmias. If within 30 days of an MI, even if the MI caused the HF, it should be categorized as a death from MI.

IV. *Death by Cerebrovascular Accident (CVA)*

It refers to death as a direct consequence of the stroke (e.g.: intracranial hypertension) or due to complications of stroke.

V. *Death by CV procedure*

It refers to deaths as a direct consequence of complications from cardiac procedures, except those used to treat coronary heart disease or MI (at which time they will be classified as death from coronary heart disease or MI, respectively).

VI. *Death due to cardiovascular hemorrhage*

Any death in the context of non-traumatic and non-procedural vascular rupture (e.g.: aortic aneurysm), hemorrhage causing tamponade (and not related to a CV procedure), and non-traumatic intracranial hemorrhage (excluding stroke cases).

VII. *Death due to other CV causes*

It refers to deaths from CV diseases not included in the previous definitions (eg: pulmonary embolism, peripheral arterial disease).

4.4.4.2. Non-cardiovascular death

It refers to deaths whose causes are not confirmed or suspected as cardiovascular. They may occur within the context of hospitalization for a CV event, but the cause of death does not involve a direct complication of the CV event (eg: a patient hospitalized for HF who dies from hospital pneumonia).

I. *Death from lung disease*

It refers to all death as a direct consequence of respiratory failure caused by a primary disease of the lung parenchyma, whether infectious or not. Excludes cases of death from pulmonary embolism or cardiogenic pulmonary edema (situations that will be considered as CV deaths).

4.4.5 Hospitalization for Unstable Angina (UA)

Hospitalization for UA will be defined as any unplanned hospitalization at the time of randomization due to an episode of precordial discomfort (or anginal equivalent), lasting ≥ 10 minutes, accompanied by at least one of the following characteristics:

Occurring at rest; OR

Occurring with decreasing levels of effort, in an accelerated pattern.

In addition, to meet the criteria for this event, there must be hospitalization within 24 hours of the last symptom, accompanied by at least one of the criteria below:

New or presumably new ST-segment or T-wave changes on resting ECG (in the absence of left ventricular hypertrophy or old LBBB), with at least 1 mm of transient ST-segment elevation, 0.05 mm of ST-segment depression or inversion of at least 0.3 mV of T-wave in leads with dominant R-wave;

Evidence of inducible ischemia in stress test performed in the same hospitalization (exercise test, bone scan, stress echocardiogram or magnetic resonance imaging);

Angiographic evidence of obstruction ≥ 70% or presence of thrombus as being responsible for the patient's symptoms;

Need for myocardial revascularization (PCI or CABG) in the same hospitalization or after subsequent transfer to another service without being discharged home.

4.4.6 Hospitalization for Heart Failure (HF)

Hospitalization for HF comprises all those situations where the patient is hospitalized for at least 24 hours with a primary diagnosis of HF and meets ALL of the following criteria:

a) The patient has at least one of the following symptoms of worsening HF: dyspnea, reduced exercise tolerance, fatigue, other symptoms of low cardiac output or volume overload (eg, ascites)

b) Objective evidence of signs of HF, through at least TWO signs on physical examination (edema; ascites; rales; hepatojugular reflux/increased jugular venous pressure; S3 gallop; rapid weight gain consistent with fluid retention) or ONE physical examination sign plus at least ONE laboratory or imaging evidence (increased atrial natriuretic peptides, radiological signs of pulmonary congestion, radiological or invasive evidence of increased filling pressures or low cardiac output)

c) The patient receives a new therapy or an increase in the previous therapy specifically for HF, such as:

- Increased dose of oral diuretic
- Intravenous use of diuretics, vasodilators or inotropic agents
- invasive therapy for HF, such as mechanical circulatory support or ultrafiltration.

If the primary cause of hospitalization is not HF, but HF is a factor that decisively contributes to the prolongation of a previous hospitalization, the event will also be positively adjudicated, provided it meets the criteria above (eg: HF complicating a hospitalization for MI).

4.4.7 Hospitalization for respiratory infections

Comprises all cases of hospitalization that have a primary diagnosis of respiratory tract infection, including tracheobronchitis, sinusitis, pneumonia, lung abscess or pleural effusion of a clearly infectious nature. The event must meet all of the following criteria:

a) Hospital stay longer than 24 hours due to the diagnosis of infection;

b) Evidence through imaging scan or clinical examination of involvement of the respiratory tract;

c) Initiation of specific antimicrobial, antifungal or antiviral therapy.

Cases of exacerbated chronic obstructive pulmonary disease with respiratory infection as a contributing cause will also be considered, provided they meet the criteria above.

If the primary cause of hospitalization is not infection, but infection is a factor that decisively contributes to the prolongations of a previous hospitalization, the event will also be positively adjudicated, provided it meets the criteria above (eg: nosocomial pneumonia complicating a hospitalization for MI).

Hospitalizations for respiratory infections will be subdivided into upper airway infections (URTI) and lung infections.

4.4.7.1 Hospitalization for upper airway infection

Comprises infections involving the ear, nose, throat, paranasal sinuses, larynx and trachea. Diagnosis can be made by evidence of clinical examination (eg: tympanic membrane bulging, posterior pharyngeal secretion, tonsil hyperemia).

4.4.7.2 Hospitalization for pulmonary infection

Comprises infections involving bronchi, bronchioles, lung parenchyma and pleural cavities. For the diagnosis of this type of infection, a compatible imaging scan is necessary (eg: chest X-ray or CT).

4.4.8 Myocardial revascularization due to ischemia

Comprises the need for myocardial revascularization (by CABG or PCI) due to findings compatible with ischemia, such as symptoms of angina (or anginal equivalent) or ischemia shown in non-invasive functional tests (eg, ergometric test) or invasive (such as fractional flow reserve – FFR). This definition includes all cases of revascularization due to an ischemic cardiac event (see items myocardial infarction and unstable angina).

The attempt to intervene in a lesion, even if unsuccessful, will be considered a revascularization event, if it meets the criteria above. In the case of percutaneous revascularization, the attempt to introduce a guidewire in order to implant any device aimed at unblocking the target vessel should be considered a revascularization event (including stent angioplasty, balloon angioplasty, atherectomy or thromboaspiration) . Isolated invasive diagnostic tests are not considered revascularization procedures, even if invasive physiology (such as FFR) or intracoronary imaging (such as intravascular ultrasound) are performed.

Myocardial revascularizations will be classified into urgent and non-urgent (elective):

I. *Urgent myocardial revascularization*

The procedure is required in a rapid period (eg, < 24 hours), usually due to an acute situation, when a delay in performing the procedure would entail a risk of deterioration in the patient's condition.

II. *Non-urgent revascularization*

The procedure is performed on an elective basis, usually in a stable condition, when the delay does not imply a risk of deterioration in the patient's clinical condition.

4.4.9 Stent thrombosis

Stent thrombosis will be an event assessed through reports of angiographic procedures. There will be no analysis of the coronary angiography image by the adjudicators. Stent thrombosis will be defined as definite, probable or possible, according to the criteria of the Academic Research Consortium (ARC). All cases of stent thrombosis must have a corresponding clinical manifestation, namely, typical symptoms of ischemia at rest, ECG changes suggesting ischemia, OR elevation of biomarkers (see criteria for myocardial infarction). In addition, the thrombus must originate within the stent or in a segment 5 mm proximal or distal to the device. Stent thrombosis will fall into one of the following categories:

A) Definite stent thrombosis: Angiographic evidence, by thromboaspiration specimen or by thrombus autopsy within a coronary stent mesh.
B) Probable stent thrombosis: Myocardial infarction in a coronary territory compatible with a vessel submitted to previous stent implantation or unexplained death within 30 days after PCI with stent.
C) Possible stent thrombosis: Unexplained death more than 30 days after PCI with stent.

4.4.10 Safety endpoints

They will be monitored after the administration of the influenza vaccine for a period of 7 days following the occurrence of adverse events, which will be reported by face-to-face or telephone interviews by the HUB investigators and described in the CRF. AEs will be classified into two groups:

(I) Local adverse events

These will be described as reactions that may occur at the vaccine administration site, such as: erythema, edema, local pain, redness, limb or site induration and

ecchymosis. These reactions are benign, self-limited, and usually resolved within 48 hours. They will all be described, if they occur, in the CRF.

(II) Systemic adverse events

These are benign and self-limited, such as fever, malaise, myalgia, hypersensitivity (anaphylactic) reactions and neurological manifestations (narcolepsy and Guillain-Barré syndrome, even without a cause-effect definition). These manifestations are more frequent in people who have not had previous contact with vaccine antigens.

Anaphylactic reactions (hypersensitivity type I) are extremely rare and can be related to any component of the vaccine. Some influenza vaccines may contain minimal amounts of egg protein, which may induce immediate allergic reactions in individuals with severe allergy to this protein. These events may be seen more frequently in individuals who have never had contact with the influenza vaccine. Fever will be classified into two groups by thermometer measurements: Fever condition will be classified when the mean body temperature presents values above 37.5 °C. Severe fever when the temperature is above 38.9 °C with or without hospitalization.

4.4.11 Seroconversion endpoint

The humoral response to vaccination will be evaluated by the hemagglutination inhibition (HAI) test. In healthy individuals, HAI titers ≥ 1:10 indicate the presence of antibodies specific to the influenza virus, and titers ≥ 1:40 indicate protection from infection by the virus components in the tests, that is, the viruses that make up the vaccine. The following definitions will be used based on the results of the HAI tests: Titers <1:10 = seronegative; Titers ≥1:40 or 4x baseline titers = protection against influenza. This 4x value will be used for assessment between the two intervention arms.

5. ADVERSE EVENTS

An Adverse Event (AE) is defined as any untoward medical occurrence, including an exacerbation of a preexisting condition, in a clinical investigation patient who received a pharmaceutical product. The event does not necessarily have to be causally related to this treatment.

5.1 Serious adverse event (SAE)

Results (defined above) that meet the criteria for a SAE will not be reported as a SAE, but as outcomes. A SAE is defined as any AE that results in death, is immediately life-threatening, results in persistent or significant deficiency/disability, requires or prolongates the patient's hospitalization, is a congenital anomaly/birth defect, or should be considered serious by any other reason if it is an important medical event when based on proper medical judgment that could jeopardize the patient and may require medical or surgical intervention to prevent one of the other outcomes listed in the definitions above.

The intensity of the AE should be judged based on the following:

- Mild: Awareness of signs or symptoms that are easily tolerated
- Moderate: Sufficient discomfort to cause interference with usual activity
- Severe: Disabling or causing inability to work or perform usual activities.

5.2 Causal relation of the adverse event

Medical judgment should be used to determine the relation, considering all relevant factors, including reaction pattern, temporal relation, withdrawal or reintroduction of study drug. Only unexpected and previously unreported serious adverse events believed to have a reasonable level of certainty to be related to study medication need to be immediately reported (i.e., within 24 hours of awareness of the event) to the VIP-ACS Project Office. For such events, the research team must complete a SAE CRF and immediately enter it into the e-CRF. The VIP-ACS Project Office will then

inform ANVISA (Brazilian Health Surveillance Agency) in a timely manner in accordance with the SAE Management Plan.

5.3 Serious Adverse Event Report

The investigator is responsible for informing local authorities and ethics committees of any unexpected serious adverse events by local requirements.

5.4 Treatment of adverse events

The treatment of any adverse event will be as identified by local investigators and based on Good Clinical Practice. The identification and therapies adopted must be described in the field specific o vaccination safety and/or in the field of Serious Adverse Events contained in the eCRF. If a serious adverse event occurs, the Study Coordinating Site must be informed.

All concomitant medications, except for vitamins and dietary supplements, must be described in the Booklet and reported in the eCRF. The use of medications for prophylaxis or control of febrile conditions must be included in the eCRF.

6. STATISTICAL ANALYSIS

6.1 Sample Size Calculation

Since the design of the original protocol, the sample size calculation was based on the combined endpoint (MACE) results from a meta-analysis of prospective randomized studies evaluating the effect of influenza vaccines in reducing cardiovascular events after a recent ACS, which revealed a 1-year reduction in relative risk of 36%.[4] The previous version of the protocol considered a conservative event reduction model due

to the use of an active control group, and an event-driven approach, estimating an event rate of 12% per year of the primary endpoint; for a superiority study and a hypothesis of relative risk reduction of 18%, for a statistical power of 90% and a 5% two-sided alpha, 953 events in approximately 9200 patients would be required to complete of the study. However, some facts impacted the VIP-ACS study. With the advent of the COVID-19 pandemic, the number of patients with ACS declined at the investigating sites, and of patients with recruitment potential, most received influenza vaccine with the same strains of the study vaccine, which deemed them ineligible to participate in the study. This fact was relevant as in Brazil the national immunization campaign against influenza was strengthened during the years 2020 and 2021, with the reduction of priority groups and focus on all individuals over 18 years of age.

Another important fact occurred in August 2021, when the IAMI trial results[50] showed that intervention with quadrivalent vaccine versus placebo in patients after ACS provided a 28% reduction in the primary composite endpoint of death from all causes, AMI and stent thrombosis. In addition, pooled data from three previous randomized studies were compiled with IAMI trial data[50] showing a 49% reduction in cardiovascular mortality with influenza vaccine versus placebo, in order to confirm previous findings derived from smaller studies with the same objective[4].

In this sense, and based on the results of the aforementioned meta-analysis, non-controllable factors (COVID-19) and the findings of the IAMI trial, we recalculated the VIP-ACS sample size using a statistical Win Ratio (WR) approach, for analysis of efficacy of the double-dose influenza vaccine compared to the standard dose vaccine, in which it was estimated that the inclusion of 1800 patients (900 per group) for a WR of 1.41 with a power of 82.6% and an alpha of 5 %. These data were obtained over 10,000 simulations, considering the following event rates in the control group: death, 7.0%; AMI, 3.4%; stroke, 1.0%; hospitalization for unstable angina, 2.7%; hospitalization for heart failure, 2.9%; emergency revascularization, 1.0% and hospitalization for respiratory infections, 1.0%, and assuming a reduction of these events with double-dose vaccine in: death by 2.0%; AMI by 0.9%; Stroke by 0.3%; Hospitalization for unstable

angina by 0.8%; Hospitalization for heart failure by 0.7%; Emergency coronary revascularization by 0.2% and Hospitalizations for respiratory infections by 0.3%.

Considering that the primary endpoint of the VIP-ACS comprises more individual endpoints than the IAMI trial[50], and from the meta-analysis[4] studies, the sample size calculation proposed to the VIP-ACS to assess possible reductions in hierarchical events of the primary endpoint is comprehensive and conservative to the purpose of the study, and overcomes the external conditions involved in the COVID-19 pandemic.

The analysis of the primary endpoint will exclude hospitalizations for respiratory causes caused by COVID-19. To this end, a sensitivity analysis and other exploratory analysis of the impact of COVID-19 on VIP-ACS events are planned.

6.2 Statistical Analysis Plan

Details of the statistical analysis plan are described in a separate document (Statistical Analysis Plan – SAP). Characteristics of the study population will be presented by group and in the total sample. Quantitative variables will be described using mean and standard deviation, median and interquartile range, minimum, maximum and number of valid observations. Qualitative variables will be presented by absolute (number of patients) and relative (percentages) frequencies. Statistical tests will not be performed to compare baseline characteristics between study groups.

The primary endpoint will be analyzed by the statistic called Win Ratio proposed by Pocock et al., (2012)[51]. The primary hypothesis will be defined as:

H0: $\Psi=1$ (win ratio = 1): The ratio of the total winning comparisons between the double-dose influenza vaccine and standard dose influenza vaccine groups is equal.

H1: $\Psi\neq1$ (win ratio ≠ 1): The ratio of the total winning comparisons between the double-dose influenza vaccine and standard dose influenza vaccine groups is different.

By this hierarchical method, all patients in the double-dose influenza vaccine group will be compared with all patients in the standard dose group. Initially, pairs will be compared for time to death truncated at 12 months. If both died, the "winner" of the comparison will be the one with a longer time between the moment of randomization and the date of death. In this same sense, the hierarchy of endpoints that make up the primary endpoint that will be evaluated after death will be AMI, stroke, hospitalization for unstable angina, hospitalization for heart failure, hospitalizations for respiratory causes and emergency coronary revascularization. If the patient is not contacted at the 12-month visit, and the follow-up time for mortality assessment is censored; paired comparisons with this individual will be carried out until the time of censoring. The other tie-breaking criteria for the events that make up the primary endpoint are described in further detail in the study Statistical Analysis Plan – SAP.

It is expected that vital conditions of all patients will be obtained during the 12-month follow-up.

Finally, we defined the test statistic (Win Ratio) as the number of wins of the pairs of each group (double-dose influenza vaccine group and standard dose influenza vaccine group).

The results will be presented with the respective 95% confidence intervals. And the final test will use a significance level of 5%.

Interaction tests will be performed for specific subgroups, including: > 60 vs. ≤ 60 years-old; male versus female; ACS presentation (with versus without increase); history vs. no history of AMI; history vs. no history of stroke; history vs. no history of heart failure; previous COVID-19 infection vs. no previous infection; history vs. no history of diabetes. Other prespecified subgroups will be detailed in the SAP.

Proportional hazards regression models for Cox survival analysis will also be performed to calculate the HR and its respective 95% CI in the assessment of the primary

endpoint. The validity of the proportional hazards assumption will be assessed using appropriate methods. If necessary, additional appropriate survival analyses will be performed as sensitivity analyses. The non-parametric Kaplan Meier method will be used to estimate the time to the first event of the primary endpoint. Secondary endpoints will be analyzed in a similar way. Additionally, a cost-effectiveness analysis will be performed using the TreeAge software.

7. FOLLOW-UP AND DATA CAPTURE SYSTEM

7.1 Follow-up

Patients will be followed for 12 months, during which they will have in-person visits upon admission/screening, in the first, sixth and 12th months. If the patient is unable to attend medical visits within the established periods, they will be contacted by telephone or videoconference by the local study teams.

7.2 Data Collection System

The means of data collection for the study are electronic medical records via web using the HIAE Data Management System. The data is entered into the system by the site team. All forms are electronically signed by the principal investigator or designee at the sites. Training and support for using the system will be made available to investigators through the coordinating site.

The aforementioned data collection and management system has been validated by previous studies and is safe and reliable for the VIP-ACS study. System features include patient registration, confidential randomization 24 hours a day, data entry, data cleaning and export for statistical analysis, event adjudication.

All patients included in the study will have their clinical data and information relevant to the study collected by qualified personnel trained for confidentiality of

information and secure storage. Volunteers who, by chance, give up their participation during the study follow-up will have their clinical data regarding the inclusion period until the date of participation withdrawal collected and stored.

HUB investigators will maintain the confidentiality of data and means of accessing the electronic platform, protecting the privacy and confidentiality of information in accordance with local regulatory bodies.

8. ETHICAL ASPECTS AND GOOD CLINICAL PRACTICE

The VIP-ACS study will be carried out in accordance with the Brazilian and international standards described in the documents below:

- Declaration of Helsinki.
- Brazilian Resolution 466/12 and related documents from the Ministry of Health.
- ICH Harmonized Tripartite Guidelines for Good Clinical Practice, 1996.

Description of ethical reasons for vaccination after discharge:

There are no guidelines or indications for the use of influenza vaccine in standard or double doses for patients following an ACS in the hospital setting. The National Immunization Program (PNI), based on the document of the World Health Organization, advises that patients with chronic heart disease constitute a priority group for influenza vaccine during campaigns, with no recommendations, guidelines or prohibitions for use out of the campaigns and primary care network settings.

Once recent data from the latest campaigns show a reduced demand for vaccination, especially priority patients, the VIP-ACS study will raise the number of vaccinated patients who will become priorities during the study follow-up.

8.1 Local Study Approval

Before starting the study, the investigator must send a copy of the research protocol, the informed consent form, and other relevant documents to the institutional Ethics Committee (EC). The EC approval letter must be sent to the Coordinating Site. Any modifications to the original protocol must also be approved by each site EC.

8.2 Informed Consent Form

All the patients screened to participate in the study must sign the Informed Consent Form before any procedure related to the study is performed.

8.3 Central Study Approval

Before starting the study, the protocol and informed consent form used at the research site and other documents must be submitted and approved by the (EC) of the Hospital Israelita Albert Einstein in accordance with local regulatory requirements.

8.4 Study Registration

The VIP-ACS study is registered at Plataforma Brasil and ClinicalTrials.gov. under NCT number 04001504.

8.5 Data Confidentiality

No patient identification data will be disclosed. The data collection system will identify patients and sites by a number. Printed medical record data will be kept confidential by all participating sites, stored in locked cabinets/drawers. The confidentiality of patients in any report and time of the study will be preserved.

8.6 Reports

The investigator must submit study progress reports to the institution's EC every six months, as well as a final report after the end of the study.

9. STUDY COORDINATION

9.1 Coordinating Site

The Coordinating Site of the VIP-ACS study will be the Academic Research Organization of the Hospital Israelita Albert Einstein. The institution is extensively experienced in the conduction of clinical research. The coordinating site team will provide training, guidance and support to participating sites to ensure compliance with the research protocol. The team has the necessary experience and level of knowledge in research methods and biostatistics, and are assisted by award-winning career researchers.

9.2 Steering Committee

Members of the VIP-ACS Study Steering Committee will be responsible for overseeing the clinical trial, including decisions to withhold or modify study procedures, if necessary, address the challenges involved in protocol implementation, review and

interpret data, and prepare the final manuscript. This Committee is presided by a Chair. Such coordination will be carried out in person or by telephone meetings held at least every three months. All other VIP-ACS study boards will report directly to the Steering Committee.

9.3 Executive Committee

It consists of the members of the Steering Committee who are part of the study coordinating site. This Committee acts as an administrative and executive arm of the Steering Committee, being responsible for operational decisions on its behalf.

9.4 Publications Committee

The members of the Executive Committee will be selected to be part of a Publication Committee, which will be responsible for writing the final manuscript and submitting it for publication. This committee will also manage the database and will be responsible for evaluating proposals for publications based on data from the VIP-ACS study.

9.5 Adjudication Process

The Clinical Events Committee (CEC) is responsible for evaluating all clinical outcomes specified in this protocol. All potential diagnoses will be entered into the CEC tracking database. There will then be an administrative review of each of the diagnoses to verify that all required documents are available. For the adjudication process, we will consider the following original documents: official medical reports regarding the event, examinations signed by a physician, and other examinations deemed relevant to the related endpoint. Electronic records or imaging scans DVD are not required, but may be

requested if official reports are not available or in case of disagreement between adjudicators or between clinical presentation and test results.

The research coordinator will prepare the necessary eCRF documents and include additional supporting information in a CEC package. HIAE will submit two copies of each outcome package to the CEC, where they will be randomly distributed to two independent medical reviewers. Alternatively, the coordinator may upload copies of these documents in the study system or send them by e-mail to the research monitor in charge for the site. Reviewers will independently review the cases assigned to them, document and provide supporting information for judgment of each case directly in the outcome package. If the two adjudicators agree, the event adjudication will be considered complete. In case of an event discrepancy between the medical reviewers, or a reviewer discretion, the case will be presented for review by at least one additional reviewer to establish ultimate adjudication. The result of the final adjudication will be entered into the database by the CEC coordinator. A copy of all signed adjudications will be displayed in each respective folder, and stored in the CEC. Additional details of specific processes for each of the two branches of the CEC will be detailed in documentation maintained separately from standard procedures at HIAE. Details of the criteria for reporting outcomes, as well as the necessary documentation, are described in a specific manual (Clinical Events Validation Manual).

9.6 Data Quality Management

Several procedures will ensure data quality, including:

A) All investigators will participate in a training session prior to the start of the study to ensure consistency of study procedures, including data collection;
B) Investigators will be able to call the study Coordinating Site to resolve issues or problems that may arise;
C) Data entry by the HIAE Data Management System is subject to various checks for open fields, plausible, possible and not-allowed value ranges, and logical checks.

The investigator who enters the data is notified of potential problems at the time of data entering;

D) Statistical techniques for identifying inconsistencies will be conducted periodically (approximately every fifteen days). Sites will be notified of inconsistencies for correction;

E) Statistical techniques for identifying fraud will be conducted periodically (every 90 days);

F) Monitoring at the sites will be carried out during the conduction of the study;

G) The Coordinating Site will review detailed reports on data screening, inclusion, follow-up, consistencies and completeness on a monthly basis. It will immediately take action to resolve any issues.

9.7 Analysis by the Independent Data and Safety Monitoring Committee

The Independent Data and Safety Monitoring Committee will have a chairman who will organize its composition by experienced professionals with the objective of evaluating the data in order to guide the efficacy and safety of the study. The committee meetings will be held in three moments: 25%, 50% and 75% of data collected to assess study status, recruitment rate, protocol adherence, data quality, loss to follow-up, specific problems with sites or other aspects that are deemed relevant. The committee will also have access to comparative data analyses between the two randomized groups, in order to monitor the safety of the studied intervention, as well as determine the effectiveness in order to suggest about possible changes or interruptions in the continuity of the study to the steering committee. The interim analyses with their respective rules for termination due to futility, harm or unequivocal benefit will be detailed in a specific document.

9.8 Study Sponsor Responsibilities

The purpose of the study is solely to obtain the best scientific knowledge in daily clinical practice, free from any conflicts of interest. The source of financial support will be recognized in presentations and publications, but it will not influence the decision to publish the results, as well as the content of the publications. The results of the VIP-ACS study will be published regardless of the positive or negative nature of the data obtained.

9.9 Responsibilities of Investigators and Sub-investigators of Participating Sites

The principal investigator of each site will conduct and/or supervise the daily operations of the study at their respective site, assisted by the sub-investigator and the research coordinators. Most tasks can be delegated by the principal investigator to team members at each research site, provided that these individuals are qualified for the tasks and properly listed on the task delegation form. However, the principal investigator will remain legally responsible for the tasks. In addition, investigators are responsible for initiating the study at their site, for maintaining study procedures, for ensuring protocol improvements, and also for ensuring data quality and veracity.

9.10 Monitoring

Representatives from the VIP-ACS project office should be allowed to visit all participating site locations periodically to assess study data, quality, and integrity. On site, they will review the study records and directly compare them to source documents and discuss the conduct of the study with the investigator, and verify that the facilities remain acceptable.

9.11 Publication of Results

The success of the VIP-ACS study will depend on the teams involved, on the efforts and collaboration of all investigators, research coordinators, and patients. Therefore, the main results will be published having as authors the Study Steering Committee on behalf of the VIP-ACS Investigators, who will be cited at the end of the article. Up to three members will be considered (at the discretion of the main investigator) per research team that participated in each phase of the study. Requests for inclusion of other names will be evaluated on an individual basis by the Publication Committee.

10. PROTOCOL AMENDMENTS

Any change to the study protocol must be registered in writing, through an amendment signed by the Principal Investigator.

EC approval and recommendation of changes is required prior to their implementation, unless there are safety reasons that outweighs the approval or recommendation.

The changes to the protocol with their descriptions regarding the amendments were carried out as recommended by CONSERVE-SPIRIT[52], described in Annex B.

11. REFERENCES

1. http://www.who.int. Website of the World Health Organization. http://www.who.int/cardiovascular_diseases/resources/atlas/en/.
2. Murray CJL, Lopez AD. Measuring global health: motivation and evolution of the Global Burden of Disease Study. Lancet. 2017;390(10100):1460-1464.
3. Murray CJ, Lopez AD. Alternative projections of mortality and disability by cause 1990-2020: Global Burden of Disease Study. Lancet. 1997;349(9064):1498-1504.
4. Udell JA, Zawi R, Bhatt DL, et al. Association between influenza vaccination and cardiovascular outcomes in high-risk patients: a meta-analysis. JAMA : the journal of the American Medical Association. 2013;310(16):1711-1720.

5. Clar C, Oseni Z, Flowers N, Keshtkar-Jahromi M, Rees K. Influenza vaccines for preventing cardiovascular disease. Cochrane database of systematic reviews. 2015(5):CD005050.
6. Lee KR, Bae JH, Hwang IC, Kim KK, Suh HS, Ko KD. Effect of Influenza Vaccination on Risk of Stroke: A Systematic Review and Meta-Analysis. Neuroepidemiology. 2017;48(3-4):103-110.
7. Vardeny O, Sweitzer NK, Detry MA, et al. Decreased immune responses to influenza vaccination in patients with heart failure. J Card Fail 2009;15(4):368-73.
8. Van Ermen A, Hermanson MP, Moran JM, et al. Double dose vs. standard dose influenza vaccination in patients with heart failure: a pilot study. Eur J Heart Fail 2013;15 (5):560-4
9. DiazGranados CA, Dunning AJ, Kimmel M,et al., Efficacy of high-dose versus standard-dose influenza vaccine in older adults. N Engl J Med. 2014;371(7):635-45.
10. DiazGranados CA, Dunning AJ, Jordanov E, et al., High-dose trivalent influenza vaccine compared to standard dose vaccine in elderly adults: safety, immunogenicity and relative efficacy during the 2009-2010 season. Vaccine. 2013;31(6):861-6.
11. Kim JH, Talbot HK, Mishina M, et al., High-dose influenza vaccine favors acute plasmablast responses rather than long-term cellular responses. Vaccine. 2016;34(38):4594-4601.
12. Lee JKH, Lam GKL, Shin T, et al., Efficacy and effectiveness of high-dose versus standard-dose influenza vaccination for older adults: a systematic review and meta-analysis. Expert Rev Vaccines. 2018;17(5):435-443.
13. Vardeny O, Udell JA, Joseph J, et al., High-dose influenza vaccine to reduce clinical outcomes in high-risk cardiovascular patients: Rationale and design of the INVESTED trial. Am Heart J. 2018;202:97-103.
14. Keller TT, van der Meer JJ, Teeling P, et al., Selective expansion of influenza A virus-specific T cells in symptomatic human carotid artery atherosclerotic plaques. Stroke. 2008;39(1):174-9.
15. Keller TT, van der Sluijs KF, de Kruif MD, et al., Effects on coagulation and fibrinolysis induced by influenza in mice with a reduced capacity to generate activated protein C and a deficiency in plasminogen activator inhibitor type 1. Circ Res. 2006;99(11):1261-9.
16. Haidari M, et al Wyde PR, Lytovsk S., Influenza virus directly infects, inflames, and resides in the arteries of atherosclerotic and normal mice Atherosclerosis 2010;208:90–96.
17. Huo C, Jin Y, Zou S,et al., Lethal influenza A virus preferentially activates TLR3 and triggers a severe inflammatory response. Virus Res. 2018;257:102-112.

18. Josset L, Zeng H, Kelly SM, et al., Transcriptomic characterization of the novel avian-origin influenza A (H7N9) virus: specific host response and responses intermediate between avian (H5N1 and H7N7) and human (H3N2) viruses and implications for treatment options. MBio. 2014;5(1):e01102-13.
19. Fröbert O, Götberg M, Angerås O, et al., Design and rationale for the Influenza vaccination After Myocardial Infarction (IAMI) trial. A registry-based randomized clinical trial. Am Heart J. 2017;189:94-102.
20. Grohskopf LA, Sokolow LZ, Broder KR, et al. Prevention and control of seasonal influenza with vaccines: Recommendations of the Advisory Committee on Immunization Practices-United States, 2018-19 Influenza Season. MMWR Recomm Rep 2018; 67:1.
21. Centers for Disease Control and Prevention. People at high risk of developing flu-related complications. http://www.cdc.gov/flu/about/disease/high_risk.htm
22. de Mello WA, de Paiva TM, Ishida MA, et al., The dilemma of influenza vaccine recommendations when applied to the tropics: the Brazilian case examined under alternative scenarios. PLoS One. 2009;4(4):e5095.
23. Alonso WJ, Viboud C, Simonsen L, et al., Seasonality of influenza in Brazil: a traveling wave from the Amazon to the subtropics. Am J Epidemiol. 2007; 165(12):1434-42.
24. Chowell G, Viboud C, Simonsen L, et al., The reproduction number of seasonal influenza epidemics in Brazil, 1996-2006. Proc Biol Sci. 2010;277(1689):1857-66.
25. Caini S, Andrade W, Badur S, et al., Temporal Patterns of Influenza A and B in Tropical and Temperate Countries: What Are the Lessons for Influenza Vaccination? PLoS One. 2016;11(3):e0152310.
26. Raboni SM, Moura FE, Caetano BC, et al., Global Influenza Hospital-based Surveillance Network (GIHSN): results of surveillance of influenza and other respiratory viruses in hospitalised patients in Brazil, 2015. BMJ Open. 2018;8(2):e017603.
27. Suh J, Kim B, Yang Y, et al., Cost effectiveness of influenza vaccination in patients with acute coronary syndrome in Korea. Vaccine. 2017;35(21):2811-2817.
28. Wilkinson K, Wei Y, Szwajcer A, et al., Efficacy and safety of high-dose influenza vaccine in elderly adults: A systematic review and meta-analysis. Vaccine. 2017;35(21):2775-2780.
29. Mombelli M, Rettby N, Perreau M, et al., Immunogenicity and safety of double versus standard dose of the seasonal influenza vaccine in solid-organ transplant recipients: A randomized controlled trial. Vaccine. 2018;36(41):6163-6169.

30. Mondini G, Braga PE, Lopes MH, et al., Prospective cohort studies to evaluate the safety and immunogenicity of the 2013, 2014, and 2015 seasonal influenza vaccines produced by Instituto Butantan. Rev Inst Med Trop Sao Paulo. 2018;60:e37.

31. Van Bellinghen LA, Marijam A, Tannus Branco de Araujo G, et al., Cost-utility of quadrivalent versus trivalent influenza vaccine in Brazil - comparison of outcomes from different static model types. Braz J Infect Dis. 2018;22(1):1-10.

32. Bacurau AG, Francisco PM. Prevalence of influenza vaccination in adults and elderly with chronic respiratory diseases. Cad Saude Publica. 2018;34(5):e00194717.

33. Sato AP, Antunes JL, Moura RF, et al., Factors associated to vaccination against influenza among elderly in a large Brazilian metropolis. PLoS One. 2015;10(4):e0123840.

34. http://www.who.int/influenza/vaccines/virus/recommendations/2019_soutemen/ . Acessado em: 01/10/2018.

35. https://www.who.int/influenza/vaccines/virus/recommendations/2020_south/en/

36. Keitel WA, Atmar RL, Cate TR, et al., Safety of high doses of influenza vaccine and effect on antibody responses in elderly persons. Arch Intern Med. 2006;166(10):1121-7.

37. Mombelli M, Rettby N, Perreau M, et al., Immunogenicity and safety of double versus standard dose of the seasonal influenza vaccine in solid-organ transplant recipients: A randomized controlled trial. Vaccine. 2018;36(41):6163-6169

38. Le Corre N, Thibault F, Pouteil Noble C, et al., Effect of two injections of non-adjuvanted influenza A H1N1pdm2009 vaccine in renal transplant recipients: INSERM C09-32 TRANSFLUVAC trial. Vaccine. 2012;30(52):7522-8.

39.https://www.fda.gov/BiologicsBloodVaccines/Vaccines/ApprovedProducts/ucm112854.htm Acessado em 18 de março de 2018.

40. http://www.in.gov.br/materia/-/asset_publisher/Kujrw0TZC2Mb/content/id/45218778/do1a-2018-10-15-resolucao-re-n-2-802-de-10-de-outubro-de-2018-45218519 - Acessado em 18 de março de 2018.

41. Chang LJ, Meng Y, Janosczyk H., et al., Safety and immunogenicity of high-dose quadrivalent influenza vaccine in adults ≥65 years of age: A phase 3 randomized clinical trial. Vaccine. 2019;37(39):5825-5834.

42. Kendal AP, Pereira MS, Skehel JJ. Hemagglutination inhibition. In: Kendal AP, Pereira MS, Skehel JJ., eds. Concepts and procedures for laboratory-based influenza surveillance. Atlanta, GA: Centers for Disease Control and Prevention and Pan-American Health Organization, 1982

43. Schaffner W, Chen WH, Hopkins RH, Neuzil K. Effective Immunization of Older Adults Against Seasonal Influenza. Am J Med. 2018;131(8):865-873.

44. Charlotte Warren-Gash, Ruth Blackburn, Heather Whitaker et al., Laboratory-confirmed respiratory infections as triggers for acute myocardial infarction and stroke: a self-controlled case series analysis of national linked datasets from Scotland. Eur Respir J. 2018;51(3).

45. Blackburn R, Zhao H, Pebody R, et al., Laboratory-Confirmed Respiratory Infections as Predictors of Hospital Admission for Myocardial Infarction and Stroke: Time-Series Analysis of English Data for 2004-2015. Clin Infect Dis. 2018;67(1):8-17.

46. Warren-Gash C, Smeeth L, Hayward AC. Influenza as a trigger for acute myocardial infarction or death from cardiovascular disease: a systematic review. Lancet Infect Dis. 2009;9(10):601-10.47. Cutlip DE, Windecker S, Mehran R, et al., Clinical end points in coronary stent trials: a case for standardized definitions. Circulation. 2007;115(17):2344-51.

48. Thygesen K, Alpert JS, Jaffe AS, et al., Fourth Universal Definition of Myocardial Infarction (2018). Circulation. 2018; 138(20):e618-e651.

49. Vardeny O, Kim K, Udell JA, Joseph J, Desai AS, et al., Effect of High-Dose Trivalent vs Standard-Dose Quadrivalent Influenza Vaccine on Mortality or Cardiopulmonary Hospitalization in Patients With High-risk Cardiovascular Disease: A Randomized Clinical Trial. JAMA. 2021 Jan 5;325(1):39-49. doi: 10.1001/jama.2020.23649.

50. Frøbert O, Götberg M, Erlinge D, et al., Influenza Vaccination after Myocardial Infarction: A Randomized, Double-Blind, Placebo-Controlled, Multicenter Trial. Circulation. 2021 Aug 30. doi: 10.1161/CIRCULATIONAHA.121.057042. Online ahead of print.

51. Pocock SJ, Ariti CA, Collier TJ, Wang D. The win ratio: a new approach to the analysis of composite endpoints in clinical trials based on clinical priorities. Eur Heart J. 2012; 33(2):176-82.

52. Orkin AM, Gill PJ, Ghersi D, Campbell L, Sugarman J, et al., Guidelines for Reporting Trial Protocols and Completed Trials Modified Due to the COVID-19 Pandemic and Other Extenuating Circumstances: The CONSERVE 2021 Statement.J AMA. 2021 Jul 20;326(3):257-265. doi: 10.1001/jama.2021.9941.

12. ANNEX A – Changes in VIP-ACS protocol

Changes in Protocol	Inclusions	Exclusions
**Version 6**	2. OBJECTIVES<br><br>2.1 Primary objective<br><br>*"Inversion of the hierarchy of primary objective, putting the endpoint for respiratory causes last"*<br><br>To assess whether increased doses of in-hospital influenza vaccine compared to outpatient vaccination in the usual dose, decrease the risk of combined cardiorespiratory events (death, AMI, stroke, hospitalization for unstable angina, hospitalization for heart failure, emergency coronary revascularization or **hospitalization for respiratory infections**) in patients with acute coronary syndrome.<br><br>4. ENDPOINTS<br><br>4.1. Primary endpoint<br><br>*"Changing the Hierarchy of Endpoints: Hospitalizations for Respiratory Infections Will be the last endpoint of the primary component."*<br><br>Combined hierarchical endpoint of death, acute myocardial infarction, stroke, hospitalization for unstable angina, hospitalization for heart failure, emergency coronary revascularization, **or hospitalization for respiratory infections**.<br><br>6. STATISTICAL ANALYSIS<br><br>6.1 Sample calculation -<br><br>*"Adjustments of study power values excluding COVID-19 cases from the endpoint hospitalizations for respiratory causes and placing it at the end of the hierarchy. "*<br><br>In this sense, and based on the results of the aforementioned meta-analysis, non-controllable factors (COVID-19) and the findings of the IAMI trial, we recalculated the VIP-ACS sample size using a statistical Win Ratio (WR) approach, for analysis of efficacy of the double-dose influenza vaccine compared to the standard dose vaccine, in which it was estimated that the inclusion of 1800 patients (900 per group) for a **WR of 1.41 with a power of 82.6% and an alpha of 5%**. These data were obtained over 10,000 simulations, considering the following event rates in the control group: **death, 7.0%; AMI, 3.4%; stroke, 1.0%; hospitalization for**	Figure 1. – Change of figure 1 to add the figure with updated primary outcome.

	**unstable angina, 2.7%; hospitalization for heart failure, 2.9%; emergency revascularization, 1.0% and hospitalization for respiratory causes, 1.0%, and assuming a reduction of these events with double-dose vaccine in: death by 2.0%; AMI by 0.9%; Stroke by 0.3%; Hospitalization for unstable angina by 0.8%; Hospitalization for heart failure by 0.7%; Emergency coronary revascularization by 0.2% and Hospitalizations for respiratory infections by 0.3%**.	
**Version 5**	**1. INTRODUCTION AND RATIONALE**<br><br>Addition of INVESTED and IAMI study results occurring previously to the preparation of this amendment.<br><br>2.1 Primary objective<br><br>Addition of respiratory events to the primary objective.<br><br>4. ENDPOINTS<br><br>4.1. Primary endpoint<br><br>Addition of endpoint: Hospitalizations for respiratory infections (excluding COVID-19 causes).<br><br>**4.3 Exploratory endpoints**<br>Occurrence of the primary endpoint between treatment groups within 45 days of randomization<br><br>6. STATISTICAL ANALYSIS<br><br>6.1 Sample Size Calculation –<br><br>Impacts could occur if the original statistical analysis plan was maintained, since the study sample would be sufficient for the previously proposed models.<br><br>In this sense, with the advent of the COVID-19 pandemic, in which there was a high rate of patients who received influenza vaccine prior to recruitment, and based on the recent results of the IAMI trial, for the study to keep its initial objective with statistical power, the study Executive Committee proposed a hierarchical statistical analysis based on Win Ratio (WR), previously (Sep. 2021) to the DSMB analyses, in which assuming that the double-dose influenza vaccine reduces Death by 1.9%; AMI by 0.9%; Stroke by 0.3%; Hospitalization for unstable angina by 0.8%; Hospitalization for heart failure by 0.9%;	Figure 1. – Change of figure 1 to add a figure with the updated primary endpoint.

	Hospitalizations for respiratory infections by 0.8% and Emergency coronary revascularization by 0.3% will be evaluated with the inclusion of 1800 patients with 81% power and 5% alpha. Therefore, it will not be necessary to include 9200 patients, or 953 events related to the primary endpoint, as proposed in version 4.0 of the protocol. The outcome Hospitalization for respiratory infections was added to the primary endpoint, since this outcome is a recurrent event in patients with HF after an ACS, and also composed the endpoint (MACE) of studies and analyzes such as: INVESTED and Udel et al; 2013 meta-analysis. Analysis of the primary outcome of VIP-ACS will exclude confirmed respiratory events from COVID-19. COVID-19 cases will be analyzed in an exploratory manner and by sensitivity tests.<br><br>6.2. Statistical analysis plan – Description of how the Win Ratio methods will be applied for the analysis of main primary and secondary endpoints.	
**Version 4**	*2.1* Primary objectives<br><br>*2.4* Exploratory objectives<br><br>*3.2.1.2* Exclusion Criterion<br><br>*Detailed description of Guillain-Barré history*<br><br>*Hospitalization length for ACS > 7 days*<br><br>3.3 Randomization Method and Confidential Allocation<br><br>*3.5* Study procedures:<br><br>*Possibility of a new inclusion wave*<br><br>*Description of the randomization up to 7 days of the index event. 30-day visit after randomization for both groups.*<br><br>*Detailed Description of Follow-up Visits*<br><br>*28-day safety visit*<br><br>*Figure 2 of the randomization and follow-up scheme*<br><br>*Modification of table 2 with description of follow-up and safety visits*<br><br>*Description of visits*<br><br>*3.6* Study Interventions<br><br>*3.82* Concurrent use of other vaccines	Figure 1 – Change of figure<br><br>Figure 4 – Change of figure<br><br>3.6 Study Interventions<br><br>Removal of trivalent vaccine<br><br>4.2 Secondary endpoints<br><br>Removal of endpoints:<br><br>Other arterial thrombotic event;<br><br>Renal endpoint.

	3.83 Vaccination during national vaccination campaigns<br><br>*3.13.1* Collection and storage procedure for biological material<br><br>4.1 Primary endpoint.<br><br>4.2 Secondary endpoints<br><br>4.3. Exploratory endpoints<br><br>4.4 Definition of endpoints<br><br>4.10 Safety endpoints<br><br>4.11 Seroconversion endpoints<br><br>*5.* Adverse events<br><br>*6.1* Sample size calculation<br><br>*6.2* Statistical Analysis Plan<br><br>7. Follow-up and data collection system<br><br>*9.5* Adjudication processes<br><br>*9.7* Analysis by the Independent Data and Safety Monitoring Committee	
**Version 3.**	Acronyms and Abbreviations (Page 07);<br><br>*2.2.* – Hypothesis (Page 15);<br><br>*3.2.1.2* – Exclusion Criteria: (Page 16);<br><br>*3.5* Study Procedures (Pages 17-20) **inclusion of vaccination periods for the years 2019-2020;**<br><br>*3.7* - Storage and use conditions of the vaccines (Page 24);<br><br>*3.8* - Adverse events and safety of vaccine (Page 25);<br><br>*3.8.1* - Local adverse events (Page 25);<br><br>*3.8.2* - Systemic adverse events (Page 25);<br><br>*3.8.3* - Safety of double use of influenza vaccine (Page 26);<br><br>3.9 - HUB-Coordinating site contact – *Helpline* (Page 26)<br><br>*3.10* - Concurrent therapies (Pages 26-27);<br><br>*3.11* - Risks and benefits to the patients (Page 29)<br><br>*3.9* - Definitions of endpoints (Pages 33 – 36);	*3.5* – Study procedures (pages 17-20) – Periods for in-hospital vaccination (up to 72 hours after the onset of ACS symptoms) and prior to PCI (up to 24 hours) were removed.

	3.13 - Data collection system (Page 35)	
**Version 2.**	*3.2.1.1* - Inclusion Criteria: (Page 15);<br>*3.2.1.2* - Exclusion Criteria: (Page 16);<br>3.5 - Study procedures: (Pages 17-18)<br>3.9 - Definitions of endpoints (Pages 21- 22)	
**Version 1.**		

13. ANNEX B. CONSERVE-SPIRIT[52]

**CONSERVE-SPIRIT Extension: October 20, 2021**			
**Item**	**Item title**	**Description**	**Page**
I.	Strenuous (exceptional) circumstances to the protocol	COVID-19 pandemic affecting patient recruitment and follow-up.	
II.	Major changes	A) Changes in the number of recruited patients and statistical analysis plan.	
		B) Impacts could occur if the original statistical analysis plan was maintained, since the study sample would be sufficient for the previously proposed models.<br>In this sense, with the advent of the COVID-19 pandemic, in which there was a high rate of patients who received influenza vaccine prior to recruitment, and based on the recent results of the IAMI trial, for the study to keep its initial objective with statistical power, the study Executive Committee proposed a hierarchical statistical analysis based on Win Ratio (WR), previously (Sep/Oct. 2021) to the DSMB analyses, in which assuming that the double-dose influenza vaccine reduces Death by 2.0%; AMI by 0.9%; Stroke by 0.3%; Hospitalization for unstable angina by 0.8%; Hospitalization for heart failure by 0.7%; Emergency coronary revascularization by 0.3% and Hospitalizations for respiratory causes by 0.2% will be evaluated with the inclusion of 1800 patients with 82.6% power and 5% alpha. Therefore, it will not be necessary to include 9200 patients, or 953 events related to the primary endpoint, as proposed in version 4.0 of the protocol. The outcome Hospitalization for Respiratory Infections was added to the primary endpoint, since this outcome is a recurrent event in patients with HF after an ACS, and also composed the endpoint (MACE) of studies and analyzes such as: INVESTED and Udell et al; 2013. Analysis of the primary outcome of VIP-ACS will exclude confirmed respiratory events from COVID-19. COVID-19	

		cases will be analyzed in an exploratory manner and by sensitivity tests.	
		c) This change occurred prior to the first assessment by the study data and safety assessment committee, in September and October 2021 the hierarchy of endpoints was reassessed, placing hospitalization for respiratory infections last based on aggregated data and assessing the study power with a relative risk reduction of 28%,, without any access to interim data by the Executive Committee, only recruitment data and aggregated reported endpoints.	
III.	People in charge for the changes	Otávio Berwanger, Henrique A. Fonseca, Remo Furtado	
IV.	Interim data	The only data used for decision making were those referring to the screening and recruitment rate, as well as the rate of aggregated events reported by the investigating sites throughout follow-up, blind to the intervention groups.	

SPIRIT Item and Number					Page
		No change	Impact*	Mitigation strategy**	
1	Title	x			
2	Study registration	x			
3	Protocol Version		Need for a new version in view of the new Win Ratio analyses for the study effectiveness	Created a new protocol version (V6.0) with the proposed analysis changes	1
4	Financing	X			
5	Roles and Responsibilities	X			
6	Rationale	X			
7	Objectives	X			
8	Study Design	X			
9	Study locations	X			
10	Eligibility criteria	X			
11	Interventions	X			
12	Endpoints		Impact in the endpoint of hospitalization for respiratory infections	Addition of the endpoint hospitalization for respiratory infections to the primary endpoint, but the causes derived from COVID-19 will not be added to the primary endpoint.	30
13	Participants follow-up	x			
14	Sample size		With the onset of the pandemic, the number of patients recruited for ACS declined over the course of the study, and most of those with ACS received previous influenza vaccine (same vaccine as the study).	Reduced to 1800 total patients (900/group), based on Win Ratio analysis estimates.	46
15	Recruitment	x			
16	Allocation	x			
17	Blinding	x			
18	Data collection methods	x			

19	Data management	x			
20	Statistical methods			Evaluation using the Win Ratio (WR) approach for analysis of the double-dose efficacy of influenza vaccine compared to standard dose vaccine will be through the inclusion of 1800 patients (900 per group) to detect a 25% reduction in the primary endpoint relative risk with a power of about 82.6% and a 5% alpha.	47
21	Data monitoring	x			
22	Study arms	x			
23	Auditing	x			
24	Ethical approval	x			
25	Protocol amendments		Need for an amendment regarding the analysis plan	Amendments 5.0 and 6.0 implemented with new analysis models (WinRatio) and number of participants	1
26	Consent	x			
27	Confidentiality	x			
28	Declaration of interest	x			
29	Data access	x			
30	Auxiliary care and post-research	x			
31	Data disclosure policy	x			
32	Informed consent form materials	x			
33	Biological samples	x			

* Uncontrolled aspects that were affected by the strenuous (exceptional) circumstances of the protocol.
** Aspects that were altered in the study by investigators, sponsors, and investors in response to strenuous (exceptional) circumstances or management that may directly impact the study.

DocuSign Envelope ID: 9B1071D4-45F6-4CD6-A552-EA21F6D1F83D

"Vaccination against Influenza to Prevent cardiovascular events after Acute Coronary Syndrome"

Evaluation of the effectiveness of double dose influenza vaccination to reduce major cardiovascular events after an acute coronary syndrome

STATISTICAL ANALYSIS PLAN (SAP)

SAP version: 2.0 dated 14 March 2022

Protocol version: 6.0 dated 20 October 2021

Declaration of Confidentiality

The information contained herein is confidential and propriety of Hospital Israelita Albert Einstein. Any unauthorized use or disclosure of this information without the priorwritten permission of Hospital Israelita Albert Einstein is expressly prohibited.

DocuSign Envelope ID: 9B1071D4-45F6-4CD6-A552-EA21F6D1F83D

ALBERT EINSTEIN
SOCIEDADE BENEFICENTE ISRAELITA BRASILEIRA

STATISTICAL ANALYSIS PLAN APPROVAL SHEET

Study title: Evaluation of the effectiveness of double dose influenza vaccination to reduce major cardiovascular events after an acute coronary syndrome

Clinical trial registry: NCT04001504

SAP Version: Draft version

SAP Version date: 14 March 2022

**Written by**	Frederico Monfardini (Statistician)	DocuSigned by: Frederico Monfardini	15/03/2022
	Name	Signature	Date
**Reviewed by**	Henrique A. R. Fonseca (Trialist)	DocuSigned by: [illegible]	15/03/2022
	Name	Signature	Date
	Remo H. M. Furtado (Trialist)	DocuSigned by: Remo Holanda de Mendonca Furtado	15/03/2022
	Name	Signature	Date

DocuSign Envelope ID: 9B1071D4-45F6-4CD6-A552-EA21F6D1F83D

SAP SUMMARY OF CHANGES

Unique SAP version identifier	Date of document version	Sections	Significant changes from previous authorized version
Version 1.0	18 January 2020		N/A - First version
Version 2.0	14 March 2022	1.1 Rationale	Addition of results from the IAM trial
		1.2 Objectives	Addition of “respiratory complications” to the primary objective of the study
		2.1 Trial design	Addition of a paragraph explaining how the trial was modified due to the COVID-19 pandemic.
		2.3 Sample size and statistical framework	Change of the primary analysis to the Win Ratio method.
		2.4 Interim analysis	Clarification that the trial did not have pre-specified stopping rules
		3.2 Adherence and protocol	Addition of a paragraph explaining how the patients in the control group

DocuSign Envelope ID: 9B1071D4-45F6-4CD6-A552-EA21F6D1F83D

		deviations	with vaccination received out of the study window due to the COVID-19 pandemic will be handled.
		5.1 Outcomes definitions	Change of the primary efficacy outcome from a composite of CV death, MI or stroke, to a hierarchical analysis by the Win Ratio method of all-cause death, MI, stroke, unstable angina, hospitalization for heart failure, urgent coronary revascularization and hospitalization for respiratory infection (excluding COVID-19).<br><br>Addition of hospitalization due to COVID-19 in the secondary outcomes.
		5.2 Analysis methods	Change of the primary analysis method from a z-test of proportions to a Win Ratio method.<br><br>Specification of the Cox proportional hazards model as a secondary analysis of the primary outcome.
		5.3 Sensitivity analyses	Addition of a sensitivity analysis including hospitalizations for respiratory infections due to COVID-19 in the primary outcome.

DocuSign Envelope ID: 9B1071D4-45F6-4CD6-A552-EA21F6D1F83D

		5.4 Multiplicity adjustments<br>5.5 Subgroup analyses	These two new section were included in the current version.
		5.6 Additional analyses	An exploratory analysis was added for respiratory outcomes.

DocuSign Envelope ID: 9B1071D4-45F6-4CD6-A552-EA21F6D1F83D

List of abbreviations and definition of terms

ACS – Acute Coronary Syndrome

AE - Adverse events

AEIH – Albert Einstein Israelite Hospital

AMI – Acute myocardial infarction

CEC – Clinical Events Committee

CI –Confidence Interval

CRF – Case Report Form

DBP – Diastolyc Blood Pressure

DD – Double Dose

ECG – Electrocardiogram

FICF – Formulary of Informed Consent Form

GBS – Guillain-Barré Syndrome

HD – Higher Dose

HR – Hazard Rate

ITT – Intention to Treat

MACE – Major Adverse Cardiovascular Events

OR – Odds Ratio

PASS – Power Analysis Sample Size

PP – Per protocol

RMST - Restricted Mean Survival Time

RR – Relative Risk

RRR – Relative Risk Reduction

SAP – Statistical Analysis Plan

SAS – Statistical Analysis System

SBP – Systolic Blood Pressure

SD - Standard Dose

TIA – Transient Ischemic Attack

VCA – Vascular Cerebral Accident

WR – Win Ratio

1. INTRODUCTION

1.1. Background and rationale

Cardiovascular diseases impose a great burden in the context of public health, especially among patients with chronic non-transmittable diseases, who have low adherence to drug therapies. Vaccination with increased dose throughout the year could be a cost-effective intervention to reduce cardiovascular events, since it is an approach of easy adherence, administration and low cost, when compared to long-standing use of medications.

A prior study demonstrated that influenza vaccination during the acute phase after myocardial infarction (MI) led to a 28% relative risk reduction in the composite of death, recurrent MI or stent thrombosis, versus placebo[1]. However, it remains unknown whether patients admitted with acute MI should be vaccinated soon after hospitalization, and whether a higher dose is more efficacious than standard dose vaccination. Therefore, a study evaluating high dose (HD) influenza vaccination in a period that contemplates the seasonality of the influenza virus in Brazil may bring important findings to different scientific gaps, as well as clarify questions about the possible benefit of double-dose vaccination, which does not present contraindications, immediately after an atherothrombotic event. If it shows real benefit, it may also be a future adjunctive therapy in the prevention of cardiovascular events.

1.2. Objectives

Primary objectives

To assess whether increased doses of influenza vaccine in the hospital phase, when compared to usual dose vaccination, reduce the risk of major cardiovascular and respiratory complications in patients with acute coronary syndrome, within 12 months of follow-up.

Hypothesis: A double dose influenza vaccination during the hospitalization phase of patients with ACS is superior in preventing cardiorespiratory events or death, compared with single dose 30 days after randomization.

DocuSign Envelope ID: 9B1071D4-45F6-4CD6-A552-EA21F6D1F83D

Secondary objectives

The secondary objectives aim to assess whether, within 12 months, in acute phase ACS patients, the increased dose influenza vaccine is superior to the routine outpatient procedure to avoid the following outcomes:

- All-cause death;
- Cardiovascular death;
- AMI (acute myocardial infarction);
- Stroke/transient ischemic attack (TIA)
- Hospitalization for unstable angina;
- Urgent myocardial revascularization;
- Non-urgent myocardial revascularization;
- Hospitalization for heart failure;
- Hospitalization for respiratory infection
- Hospitalization for COVID-19 or death for COVID-19;
- Stent thrombosis.

Additional objectives

Assess whether there is an interaction between the effect of the vaccine in the acute phase and influenza season in the southern hemisphere.

Sub study objectives of immunogenicity, biomarkers and virology assessments

The VIP-ACS study will select centers in different regions of viral seasonality to evaluate:

- The correlation between the seroconversion to vaccination and the age of patients (> 65, ≤ 65 years)
- Whether the increased seroconversion through the use of an increased dose of the vaccine may have implications for the primary outcome of the study.
- The correlation of the findings of the study to immune responses following vaccination, via cytokines, or other biomarkers.
- The correlation between the viral typing of the patients included in the study and the type of outcome, since certain viral subtypes may be more immunogenic.

DocuSign Envelope ID: 9B1071D4-45F6-4CD6-A552-EA21F6D1F83D

2. STUDY METHODS

2.1. Trial Design

A randomized controlled, open-label, multicenter, superiority, parallel group and 1:1 allocation trial, with blind assessment of clinical outcomes.

After evaluation of an acute coronary syndrome (ACS) confirmed by exams, and during hospitalization phase, if the patient attends to the enrollment criteria and signs the consent form, the randomization to one of the vaccination arms of the study will occur.

The patient can be randomized to one of the following arms:

1) Double-dose influenza vaccination (DD) – administered during the hospital stay phase, as early as possible following randomization

2) Standard dose influenza vaccination (SD) – administered 30 ± 5days after randomization Subsequently to the randomization date, each patient will be followed for 12 months, for the monitoring of cardiovascular and respiratory events.

The study was planned to initiate the randomizations from the availability of the 2019 influenza vaccine (March 2019) and continue until November 2020, even beyond the national influenza immunization campaign period for those years. Despite the effort made to completion of the planned inclusion sample, ensuring the representativeness of both planned influenza periods, the first recruitment period was impaired due to regulatory delays in the study initiation. Since the beginning of the second recruitment period, the protocol was amended (version 4, of 04-Jul-2020), the study sample size was recalculated as an event-driven study, and the timing of final analysis was set as the date when 953 primary efficacy outcome events would have occurred, even if it was necessary to continue recruitment through the year 2021.

In 2020, the COVID-19 pandemic impacted recruitment and conduct of the trial. First of all, national influenza vaccination campaign was anticipated and the adherence to immunization was higher than before, making fewer patients eligible for the study during this recruitment period. Second, enrollment was hurdled because patients were constrained of returning to study in-person visits to receive the outpatient vaccination, should they have been assigned to the control group. Due to those issues, the study steering committee decided to end recruitment after 1,800

DocuSign Envelope ID: 9B1071D4-45F6-4CD6-A552-EA21F6D1F83D

patients from an initial sample size of 9000 have been enrolled. This decision was made without any knowledge of data comparing the study groups by the steering committee or by external independent committee. At that time, the sample size was recalculated, changing the analytical method of the primary endpoint from a z-test with first event from a composite outcome to a Win Ratio approach, and including all-cause mortality and hospitalization for respiratory infections (excluding COVID-19) in the primary endpoint. With the participants enrolled at the time and considering the aggregated reported event rates, the study would preserve a power of > 80% for detecting a relative risk reduction of 28% in the components of the primary endpoint, therefore compatible with a recently published randomized trial in the field and a meta-analysis of previous trials suggesting that magnitude of effect as being plausible and clinically relevant[1,3]. After this decision, the protocol was amended (version 6.0, of 20-Oct-2021) and a new version of the statistical analysis plan was drafted.

2.2. Randomization

The randomization was performed through the electronic CRF, using the HIAE Data Management System, which was validated by previous studies, presenting safety and reliability for the VIP-ACS study. The randomization list of patients was generated considering a random function with equal probability of allocation to one of the vaccine therapies groups, stratified by research center, using blocks of 8, 10 and 12. Each vaccination arm was numbered and only the numbers were used in the randomization, which was done by the central electronic randomization of the project's coordinating center in the HIAE, ensuring the concealed allocation.

This is an open-label study in which researchers, health care workers and patients are aware of the treatment allocation. All clinical outcomes were evaluated and validated by an Independent Clinical Events Committee, whose members were blinded to the study arm assignments.

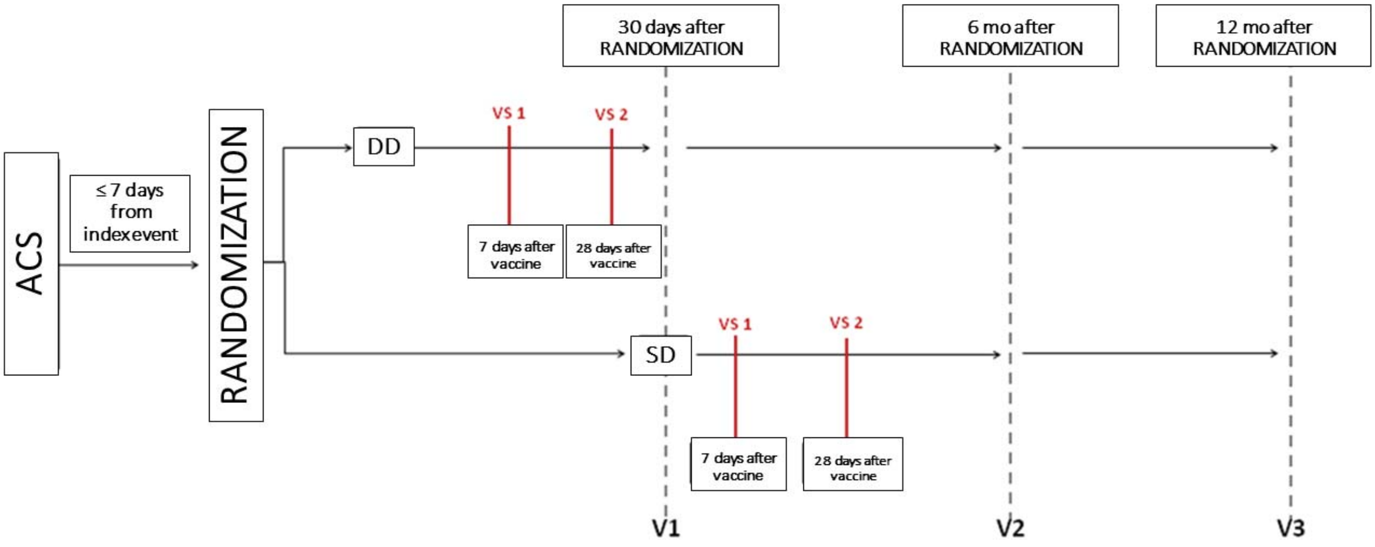

Figure 1. Randomization scheme and follow-up of the VIP-ACS study. ACS = acute coronary syndrome; DD = Double dose of vaccine; SD = Standard dose; V1 = visit 1; V2 = visit 2; V3 = visit 3; VS 1 = visit for safety 1; VS 2 = visit for safety 2; mo= months.

2.3. Sample Size and Statistical Framework

The sample size calculations were based on the primary efficacy outcome, that is, the hierarchical analysis time to of all-cause death, myocardial infarction, stroke, unstable angina, hospitalization for heart failure, urgent coronary revascularization and hospitalization for respiratory infection (excluding COVID-19), using the win ratio method[Erro! Fonte de referência não encontrada.]. In the win ratio method, every patient from the intervention group will be compared against every patient from the control group. Among each unique patient pair, the “loser” will be the first patient who presents an outcome, according to a predefined hierarchy. For example, if patient X dies and patient Y is alive by the end of follow-up, patient Y will be considered the “winner” in that match. If both participants die during the study, the “winner” will be the participant with the longest time alive since randomization. In case the patients remain tied – if neither dies by the end of 12 months or if both die with the same follow-up time – the next item in the hierarchy, myocardial infarction, will be analyzed in the same

manner (presence vs. absence of event and, if both presented the outcome, time to first event). If the patient pair remains tied, the analysis will be proceeded similarly for each component of primary outcome in the hierarchy. A tie will be declared if participants remain tied after the final item, hospitalization for respiratory infection. This will happen if neither patient presents an outcome or if they present the same outcomes for the first time at the same follow-up times. Participants will be censored at the 12-month follow-up; if the follow-up time for any participant is censored before this time due to any reason, comparisons with this individual will be made only until the moment of censorship.

The win ratio method will be analyzed as the following:
H0: Ψ=1 (win ratio = 1): the ratio of all winners in the intervention group by all winners in the control group is equal to 1.
H1: Ψ≠1 (win ratio ≠ 1): the ratio of all winners in the intervention group by all winners in the control group is not 1.

The win ratio statistic is defined as the ratio of wins (N_w: Number of Wins of the intervention group, N_L: Number of Losses of the intervention group) in each group (intervention vs. control).

$$Win\,ratio = \frac{\sum N_w}{\sum N_L}$$

This result will be presented with the accompanying 95% confidence interval. The p-value will be obtained by the following formula, as described by Pocock et al. (2012):

$$z = (P_w – 0.5) / [P_w*(1–P_w) / (N_w + N_L)]^{1/2}$$

Where N_w indicates the number of wins, N_L indicates the number of losses, and P_w indicates the proportion of wins: $P_w = N_w / (N_w + N_L)$.

Based on the trial´s reported aggregate data by the time the second enrollment season ended, we have calculated the sample size considering the following control-group estimated rates based on events-reported (non-adjudicated) at 12 months for the components of the hierarchical endpoints:

DocuSign Envelope ID: 9B1071D4-45F6-4CD6-A552-EA21F6D1F83D

Expected event rates in the control arm

Death	7.0%
Myocardial Infarction	3.4%
Stroke	1.0%
Unstable angina	2.7%
Hospitalization for heart failure	2.9%
Urgent revascularization	0.6%
Hospitalization for respiratory infection (Excludes COVID-19)	1.0%

Expected absolute risk reductions for the components of the primary outcome (representing 28% relative risk reduction)

Death	2.0%
Myocardial Infarction	0.9%
Stroke	0.3%
Unstable angina	0.8%
Hospitalization for heart failure	0.8%
Urgent revascularization	0.2%
Hospitalization for respiratory infection (Excludes COVID-19)	0.3%

Sample size and power calculation were based on 10,000 simulations. Based on these aggregated data and assuming independence between events, Bernoulli distribution for event occurrence, exponential distribution of time to outcome, and a 28% relative risk reduction (RRR) with high dose group versus standard dose group, for a two-tailed alpha = 0.05, we calculated that the study would have 82.6% power with 1800 patients enrolled (900 per group). This definition of RRR will be a conservative estimate based on a prior meta-analysis suggesting RRR of 48% with the influenza vaccination versus placebo among patients with ACS[Erro! Fonte de referência não encontrada.] and similar to recent IAMI trial a MACE reduction in 28% in ACS patients using influenza vaccine. Of note, although in the IAMI trial the intervention group (in-hospital vaccination after ACS) was compared against placebo, most of the benefit occurred almost

DocuSign Envelope ID: 9B1071D4-45F6-4CD6-A552-EA21F6D1F83D

immediately, with great separation of event curves within the first 45 days, when the incidence of cardiovascular outcomes is highest. Since we are also comparing the earliness of influenza vaccination, we also expect this magnitude of benefit to be reasonable in our study.

2.4 Statistical interim analyses and stopping guidance

The study did not have any pre-specified stopping rule for efficacy or safety.

2.5 Timing of outcome assessments

The primary and secondary outcomes will be recorded in e-CRF at the three follow-up visits: 30 days from hospital discharge (± 5 days), 6 months (± 10 days) and 12 months (± 20 days) from randomization. Detailed information on the outcomes will be collected, including the date and time of outcome occurrence, at such visits.

Regarding the vaccine effects, all patients were evaluated for a period of 7 consecutive days after the vaccination date, by hospital interview or by phone, in order to evaluate and register local and systemic adverse events that may occur. The schedule of study procedures is given in the Table 1.

Table 1 - Description of the procedures and visits of the VIP-ACS study

	**Hospital period**	**30 days from Hospital discharge**	**6th month of inclusion**	**12th month of inclusion**
Assessments				
Screening/Randomization	X			
Eligibility Criteria Review	X			
Previous medical history	X			
Socio-demographic data	X			
Medications in use	X	X		
FICF	X			
**Clinical Events Evaluation**		**X**	**X**	**X**
**Physical Measures**				
Blood pressure, pulse, and vital data	X			
Physical examination	X			
Anthropometry	X			

**Study Intervention**				
Vaccination	X	X		
Evaluation of safety outcomes (7 days after vaccine)	X	X		

3. STATISTICAL PRINCIPLES

3.1. Confidence intervals and P values

Significance testing will be 2-tailed using α = 0.05, unless otherwise specified. All analyses and summaries will be produced using R software, version 4.0.3 (or higher), or SAS® version 9.4 (or higher). Confidence intervals, if applicable, were calculated at the 95% nominal confidence level (95% CI).

The primary efficacy outcome will be tested at 5% two-tailed significance level. The second outcomes family were tested following a fixed-sequence method [please, see section 5.4]. A fixed-sequence statistical strategy tests endpoint in a predefined order (see section 5.4), all at the same significance level alpha (α = 0.05). If an individual test during any step is not statistically significant, further treatment comparison may continue (i.e., reporting of p-values) but significance will not be claimed, and p-values were deemed as nominally significant.

3.2. Adherence and protocol deviations

As this study does not contain administration of drugs, except the initial dose of vaccine, the extent of exposure does not apply. The adherence to study will be evaluated in terms of loss of follow-up, as presented in Section 4.4.

A protocol deviation is any noncompliance with the clinical study protocol, Good Clinical Practice, or Manual of Procedures requirements. The noncompliance may be on the part of the subject, the investigator, or study staff.

Major protocol deviations will be summarized by treatment group for all randomized patients. The categories of protocol deviations will include the following:

- Entered but did not satisfy enrollment criteria
- Received a disallowed concomitant treatment
- Received wrong treatment or incorrect dose

DocuSign Envelope ID: 9B1071D4-45F6-4CD6-A552-EA21F6D1F83D

- Developed withdrawal criteria but not withdrawn
- Other (as appropriate)

Investigators may only implement a deviation from or a change to the protocol to eliminate an immediate hazard(s) to subjects.

The number (and percentage) of patients with major and minor protocol deviations will be summarized by treatment group with details of type of deviation provided. The patients that are included in the Intention to treat (ITT, see section 3.3) analysis data set will be used as the denominator to calculate the percentages. No formal statistical testing will be undertaken.

Due to the COVID-19 pandemic, some patients from the control group were expected to receive study vaccine out of their expected window according to the protocol. Constraints regarding transmission of COVID-19 among participants should they leave their homes to be vaccinated are a major reason for this concern. Therefore, there will be sensitivity analysis excluding patients from the control group who were not vaccinated within the protocol window of time (i.e., 30 ± 5 days after randomization).

3.3. Analysis populations

The following analysis populations will be defined for statistical analysis:

Screened – All patients screened for the study – having an acute coronary syndrome (ACS) during the hospitalization phase.

Eligible but not randomized – All screened patients who were eligible but not randomized due to the inclusion/exclusion criteria of the study.

Intention to treat (ITT) – All eligible patients who received a randomization number, and analyzed according to the group to which they were assigned, regardless of receiving the vaccine or not.

Per Protocol (PP) – All ITT patients who did not have major protocol violation. A protocol violation means a patient who was randomized but was not eligible (had not inclusion criteria or had at least one exclusion criterion); a patient who was treated with an immunization different from the one the patient was randomly assigned (e.g., intervention arm being

DocuSign Envelope ID: 9B1071D4-45F6-4CD6-A552-EA21F6D1F83D

vaccinated after discharge). Patients vaccinated out of the window in the control arm due to the COVID-19 pandemic will still be included in the PP population, but a sensitivity analysis will be done excluding those patients as well (modified PP).

Modified PP –Due to the COVID-19 pandemic, some patients from the control group were not vaccinated within the expected study window or were vaccinated with a vaccine other than the study vaccination. Therefore, a modified PP population excluded these patients, as well as those ones that were not vaccinated at all (except for deaths occurred before vaccine could be administered)

Safety - All patients who received the vaccine. Patients who were vaccinated out of window or with vaccines other than study immunization (e.g., from an outpatient clinic) due to the COVID-19 pandemic were included in this analysis. Patients were analyzed according to the vaccination arm received, regardless of the randomized arm.

To avoid bias, if a patient had entered into study more than once, and in all these entries, this patient was considered eligible (i.e. met all inclusion/exclusion criteria), only the "first" enrollment will be maintained at the "Eligible patient population", and the other(s) were excluded, due to duplicity.

The primary efficacy analysis of the study comprises the ITT population. Safety analysis was made for the Safety population. Efficacy endpoints were performed in the PP and modified PP populations, and safety endpoints in the ITT population, as sensitivity analyses.

4. TRIAL POPULATION

4.1. Screening data

The patient disposition tables will summarize the following:

1) For recruitment period and overall:

- The number of patients screened
- The number of patients who failed screening (non-eligible) - % calculated from the Screened population
- The number (%) of eligible patients into the study (% calculated from the Screened population) and the reasons for non-eligibility

2) For each randomization group and overall:

DocuSign Envelope ID: 9B1071D4-45F6-4CD6-A552-EA21F6D1F83D

- The number (%) of randomized patients (ITT population) by site study
- The number (%) of randomized patients (ITT population) by site region and state
- The number (%) of patients in the different study populations (Eligible, Safety, ITT, PP and modified PP populations).
- The number (%) of patients who completed the study
- The number (%) of patients who withdrew from the study and associated reasons (% calculated from the Safety population)

The percentages will be calculated based on the number of randomized patients (ITT population), unless otherwise specified.

4.2. Eligibility

Inclusion Criteria:

- Men and women aged ≥ 18 years;
- Patients hospitalized with an acute coronary syndrome

Exclusion Criteria:

- Participation in another clinical trial;
- Refusal to provide consent for the study;
- Hypersensitivity or anaphylaxis to any component of the vaccine;
- History of Guillain-Barré Syndrome within the six weeks after previous influenza vaccination;
- Have already received the influenza vaccine with the same strains used in the study within 12 months of inclusion in the study (it comprises trivalent or tetravalent vaccines with the same strains of the study);
- Pregnant women;
- Breastfeeding women;
- Presenting ACS between the months of December and February.
- Time from hospitalization for ACS > 7 days.

DocuSign Envelope ID: 9B1071D4-45F6-4CD6-A552-EA21F6D1F83D

4.3. Recruitment

A CONSORT flow diagram will be used to summarize the number of patients who were:

- Assessed for eligibility at screening
- Screened
- Ineligible at screening*
- Eligible and randomized (ITT population)
- Eligible but not randomized*
- Loss of follow-up*
- Randomized and included in the primary analysis
- Randomized and excluded from the primary analysis*

*reasons will be provided.

4.4. Withdrawal/follow-up

The numbers of losses to follow-up (drop-outs and withdrawals) over the course of the trial will be summarized by treatment group using tables.
The reasons will be listed by patient and group.

4.5. Baseline patient characteristics

Patients will be described with respect to the type of ACS event at the time of selection (STEMI, NSTEMI or unstable angina), and demographic data (age, sex, race); lifestyle (smoking); medical history and comorbidities (arterial hypertension, dyslipidemia, diabetes, previous acute myocardial infarction, previous coronary angioplasty, previous surgical coronary revascularization, prior stroke, heart failure, creatinine clearance, history of hospitalization for influenza; and interventions performed for the index event before randomization (thrombolysis, percutaneous coronary intervention, myocardial revascularization surgery).

Continuous variables will be described by mean ± standard deviation or median (interquartile range), according to distribution. Categorical and ordinal data will be summarized using the number and percentage of patients. Patients with missing data will not be included in calculations of percentages, unless otherwise specified. When relevant, the number of

DocuSign Envelope ID: 9B1071D4-45F6-4CD6-A552-EA21F6D1F83D

patients with missing data will presented. All baseline characteristics will be summarized overall and separately for the two randomized groups.

5. ANALYSIS

5.1. Outcome definitions

Any suspected cardiovascular event suggestive of an endpoint as well as all deaths were submitted to the blind independent Clinical Events Committee (CEC). The CEC reviewed the data of the reported cases in a blinded manner for adjudication purpose and validate if the event should be considered as an endpoint. Cardiovascular and respiratory events adjudicated and validated by the CEC will be used for the analyses.

Events that the CEC could not classify as well as suspected event according to investigator but not confirmed by the CEC will not be part of the outcomes. defined in study protocol, the following outcomes will be evaluated:

Primary efficacy outcome: A hierarchical outcome consisting of all-cause death, myocardial infarction (MI), stroke, unstable angina, hospitalization for heart failure, urgent coronary revascularization or hospitalization for respiratory infection (excluding COVID-19).

Key secondary outcome: A hierarchical outcome consisting of CV death, MI, or stroke.

Secondary efficacy outcomes:

The first occurrence of the following endpoints:

- Total mortality
- CV mortality
- Myocardial infarction
- Stroke
- Hospitalization for unstable angina
- Need for myocardial revascularization (urgent and non-urgent)
- Hospitalization for heart failure
- TIA (Transient ischemic attack)
- Hospitalization for respiratory infections (including COVID-19)
- Hospitalization for respiratory infections (excluding COVID-19)
- Hospitalization for COVID-19

DocuSign Envelope ID: 9B1071D4-45F6-4CD6-A552-EA21F6D1F83D

- Stent thrombosis

Additionally, the primary efficacy outcome with events truncated at 45 days after randomization (i.e., the period of maximum exposure for the intervention group) will also be analyzed.

Safety outcomes:

- Number and percent of unexpected serious adverse events (event that presents immediate risk to life, persistent or significant disability, requiring or prolonging the hospitalization, resulting in congenital anomaly, Guillian Barrè syndrome, anaphylactic vaccination reaction, Peripheral Facial Paralysis, encephalitis or myelitis, optic neuritis, Stevens-Johnson syndrome, toxic epidermal necrolysis, anaphylactic vaccination reaction, another unexpected event judged as serious).
- Local and systemic adverse events after vaccination.
- Use of other vaccine than those available in the study (Hepatitis, Trivalent Influenza (Government), Pneumococcal, Yellow fever, Viral triple, DPT Adult, HPV).

5.2. Analysis methods

Primary efficacy outcome: The analysis of primary efficacy endpoint–a hierarchical outcome consisting of all-cause death, MI, stroke, hospitalization for unstable angina, hospitalization for heart failure, urgent coronary revascularization or hospitalization for respiratory infection (excluding COVID-19) will be the comparison between the two arms using the unmatched win ratio method (refer to section 2.3).

Secondary key efficacy outcome: The secondary key outcome is defined also as a hierarchical outcome of CV death, MI, or stroke, in that order, and will be compared with the Win Ratio statistic using the same definitions for the primary endpoint.

Secondary efficacy outcomes: The analysis of the secondary efficacy endpoints, defined as time to first event of: All-cause mortality, Mortality from cardiovascular causes, Myocardial infarction, Stroke, Hospitalization for unstable angina, Need for myocardial revascularization (urgent and non-urgent), Hospitalization for heart failure, TIA (Transient ischemic attack), Hospitalization for respiratory and pulmonary infections, Hospitalization for COVID-19, and Stent thrombosis; will be tested using Cox proportional hazard models. All the effects statistic

DocuSign Envelope ID: 9B1071D4-45F6-4CD6-A552-EA21F6D1F83D

will be Hazard Ratio (HR) between the two arms within 12 months, and corresponding 95%CI will be provided. Underlying assumptions of the Cox Proportional Hazard Model will be checked using graphical methods

The estimates of time to the first event (median time of event-free) will be also computed if possible, and Kaplan-Meier curves will be displayed, by treatment arm. Additional efficacy outcomes (Influenza season in the southern hemisphere): the prevalence of each strain within of the subtypes circulating in each season and location will be described in tables using proportions and graphically. The proportions will be compared using comparison of proportions tests.

Safety Outcomes: The number of patients having local and systemic adverse events up to 7 days after vaccination will be described by group and overall. The number or patients having any vaccination adverse event will be computed, and the 95% CI will be presented.

All the adverse events will be listed, by patient.

5.3. Sensitivity analyses:

A sensitivity analysis will be performed for the primary efficacy outcome including also hospitalizations for respiratory infections due to COVID-19.

Additionally, the primary and key secondary combined outcomes will also be evaluated by survival Cox proportional hazard models considering the first occurrence of any of the events listed. This approach does not define any hierarchy among the events.

5.4. Multiplicity adjustments

In order to keep the trial wise type I error, a closed test hierarchical approach will be used for adjustment for multiple hypothesis. If a difference at a significance level of 0.05 is found after a non-significant p-value, that test will be declared only nominally significant, and any further finding in the hierarchical sequence will be deemed as only exploratory. First, the primary outcome will be tested, followed by the key secondary outcome, then the following secondary outcomes, in this respective order: all-cause mortality, mortality from cardiovascular causes, myocardial infarction, stroke, hospitalization for unstable angina, need for myocardial revascularization (urgent and non-urgent), hospitalization for heart failure, TIA (transient ischemic attack), hospitalization for respiratory and pulmonary infections, hospitalization for covid-19, and stent

DocuSign Envelope ID: 9B1071D4-45F6-4CD6-A552-EA21F6D1F83D

thrombosis. For this framework, both primary and key secondary outcomes will be analyzed by the Win Ratio method, while the other secondary outcomes will be analyzed by the Cox proportional hazard models with HR and 95% CI.

5.5 Subgroup analyses:

As many of the baseline characteristics are known to be prognostic for the clinical outcomes in the study population with high-risk cardiovascular disease (as sex, age group, race/ethnicity, smoking status, etc.), internal consistency of the primary analysis will be assessed in subgroups defined by these and other baseline characteristics.

Heterogeneity of efficacy will be assessed using interaction tests of treatment by each of these baseline covariates and by influenza season (Period), using the same aforementioned models.

The following subgroups will be prespecified in this SAP:

- Male vs. females;
- Age ≥ vs. < 60 years;
- ACS presentation: (STEMI, NSTEMI, unstable angina)
- Diabetes vs. no diabetes;
- Smokers vs, non-smokers
- White race vs. non-white
- Killip class 2 or more vs. Killip class 1
- Previous heart failure vs not;
- Previous MI vs not;
- Previous stroke vs. not;
- Previous PCI vs not;
- Previous CABG vs not;
- History of chronic lung disease vs not;
- Prior COVID-19 vs.not.

- Time from presenting ACS to randomization (in tertiles)
- Time from hospitalization to randomization (in tertiles)
- Influenza season (Mar-Jul vs. other months);
- Enrollment year (2019 vs. 2020);
- Regions of the country (North and Northeastern vs others)
- COVID-19 vaccination vs not-vaccination.

5.5. Missing data

For categorical variables, patients with missing data will not be included in calculations of percentages, unless otherwise specified. When relevant, the number of patients with missing data will be presented.

All missing or partial data will be presented in the subject data listing as they are recorded on the CRF including best estimate dates of site investigators (see below) collected in the clinical database. All efforts will be made to collect complete data for all subjects randomized in this study.

Missing or incomplete event dates

All time variables, such as age, time to/from event or duration, will be calculated as: Date 2 (Final) – Date 1 (Initial) + 1. If the variable is calculated in months, the time in days will be divided by 30.44; if in years, the time will be divided by 365.25.

When an event date is unknown, the site investigator will be asked to provide a best estimate as to when the event may have occurred. Even though the exact date of an event is unknown, the investigator often does know some information that would indicate the approximate date, such as the first week of a month, in the fall of a year, or the middle of a particular year, or at least the date when the subject was last seen or contacted. This information can be meaningfully incorporated into the estimated date recorded, as this is likely to be closer to the true date than any produced by an uninformed computer program. This estimated date should be the middle date within the period that the event is known to have occurred. If the event is known to have occurred in the first week of a month, then the date in the middle of that week should be recorded as the estimate. If it occurred in the fall of a year, then the

middle date in the fall is the appropriate estimate. If no information is known then the date in the middle of the plausible time period should be given, based on the last contact with the subject prior to the event and the date of contact when information about the event was known. This method for date estimation has been used in many studies and is recommended by Dubois and Hebert (2001) [4].

If the site investigator does not provide a best estimate as to when the event occurred, the study team will follow the above rules to estimate the event date. If the date/time information is not sufficient to determine whether an event occurred prior or after randomization, the event is considered as an outcome, to be conservative. The event start date will be imputed no earlier than randomization date.

Missing data related of unobserved follow-up time up until the timing of final analysis

Unobserved follow-up time may occur due to subjects who are non-compliant with study follow-up, for example due to loss of follow-up or premature complete withdrawal of informed consent. Subjects censored administratively at the end of follow-up period or censored at time of non-CV death (in the case of the key secondary outcome) are not contributing missing follow-up time.

Missing data due to follow-up non-completion before experiencing a primary efficacy outcome event will be addressed by assuming that the data is missing at random (MAR assumption).

The sensitivity analysis of the potential impact of missing data on the primary efficacy outcome follows the elements described by Little et. al. (2016), and involves two steps:

1. A descriptive comparison of key baseline characteristics and post-randomization events preceding the end of follow-up to assess whether subjects with missing data differ systematically from subjects who complete the follow-up.

2. A pattern mixture model using multiple imputation techniques to investigate the potential impact of missing data on the primary efficacy analysis if non-ignorable censoring is assumed to be differential.

In addition, the extent of missing data will be described by the fraction of subjects with unobserved follow-up time and the fraction of unobserved follow-up subject-years.

All analyses of the potential impact of missing data will be performed in the ITT analysis set.

5.6. Additional analyses

Recurrent events analysis will be performed for the following secondary outcomes: Myocardial infarction, Stroke, Hospitalization for unstable angina, myocardial revascularization (urgent and non-urgent), Hospitalization for heart failure, TIA (Transient ischemic attack), Hospitalization for upper respiratory and pulmonary infections, and Stent thrombosis, using the Andersen and Gill model.

A pre-specified analysis will be done for infectious outcomes, with the following exploratory endpoints: all cause death or hospitalization for respiratory infections; death for pulmonary or infectious causes or hospitalization for respiratory infections; death or hospitalization for COVID-19. Same analytical methods mentioned before will be applied in these analyses.

5.7. Harms

Serious adverse events that are possible trial endpoints will be analyzed using the methods planned to the primary and secondary outcomes.

The occurrence of adverse events will be monitored for a period of 7 and 28 days following the administration of the influenza vaccine, which will be reported by face-to- face or phone interviews by site staff and described in the CRF. AEs will be classified into two groups:

a) Local adverse events

They will be described as reactions that may occur at the site of application of vaccines, such as: erythema, swelling, local pain, redness, limb or local hardening and ecchymosis. These benign reactions are usually self-limited and resolved within 48 hours. All will be described, if they occur, in the CRF.

b) Systemic adverse events

They are benign, self-limited, such as fever, discomfort, myalgia, hypersensitivity reactions (anaphylactic) and neurological manifestations (Narcolepsy and GBS, even without cause-effect definition). These manifestations are more common in people who have not had prior contact with the vaccine antigens.

DocuSign Envelope ID: 9B1071D4-45F6-4CD6-A552-EA21F6D1F83D

Anaphylactic reactions (type I hypersensitivity) are extremely rare and may be related to any component of the vaccine. Some influenza vaccines may contain minimal amount of egg protein and may induce immediate allergic reactions in individuals with a severe allergy to this protein. These events may be more noticeable in individuals who have never had contact with the influenza vaccine.

Fever will be classified into two groups by thermometer measurements:

a) Will be classified as fever when average body temperature values are higher than 37.5 °;

b) Will be classified as severe fever when the temperature is higher than 38.9 ° with or without hospital admission.

6.0 STATISTICAL SOFTWARE

Sample size calculation was made using R software, version 4.0.3.

REFERENCES

1.Frøbert O, Götbeg M, Erlinge D, Akhtar Z, Christiansen EH, MacIntyre CR, Oldroyd KG, Motovska Z, Erglis A, Moer R, Hlinomaz O, Jakobsen L, Engstrøm T, Jensen LO, Fallesen CO, Jensen SE, Angerås O, Calais F, Kåregren A, Lauermann J, Mokhtari A, Nilsson J, Persson J, Stalby P, Islam AKMM, Rahman A, Malik F, Choudhury S, Collier T, Pocock SJ, Pernow J. Influenza Vaccination after Myocardial Infarction: A Randomized, Double-Blind, Placebo-Controlled, Multicenter Trial. Circulation. 2021; 144: 1476–1484.

2. Redfors B, Gregson J, Crowley A, McAndrew T, Ben-Yehuda O, Stone GW, Pocock SJ. The win ratio approach for composite endpoints: practical guidance based on previous experience. Eur Heart J. 2020; 41 (46):4391-4399.

3. Udell JA, Zawi R, Bhatt DL, Keshtkar-Jahromi M, Gaughran F, Phrommintikul A, Ciszewski A, Vakili H, Hoffman EB, Farkouh ME, Cannon CP. Association between influenza vaccination and cardiovascular outcomes in high-risk patients: a meta-analysis. JAMA. 2013; 310 (16):1711-20.

4. Dubois MF, Hébert R. Imputation of missing dates of death or institutionalization for timeto- event analyses in the Canadian Study of Health and Aging. Int Psychogeriatr., 2001; 13 Supp 1:91-7
