# Supplementary material for: COVID-19 Vaccination and Cardiopulmonary Events After Acute Coronary Syndromes: A Secondary Analysis of a Randomized Clinical Trial
Source: JAMA Netw Open. 2024 May 30;7(5):e2413946. doi: 10.1001/jamanetworkopen.2024.13946 (PMC11140521; doi:10.1001/jamanetworkopen.2024.13946)
Supplement: Supplement 3. — Data Sharing Statement [file jamanetwopen-e2413946-s003.pdf]

**Data Sharing Statement**

Fonseca. COVID-19 Vaccination and Cardiopulmonary Events After Acute Coronary Syndromes: A Secondary Analysis of a Randomized Clinical Trial. *JAMA Netw Open*. Published online May 30, 2024. doi:10.1001/jamanetworkopen.2024.13946

**Data**
**Data available:** No

**Additional Information**
**Explanation for why data not available:** Sharing of the data would not be available since the consent was not obtained for data sharing.
